# Aptamers as Diagnostic and Therapeutic Agents for Aging and Age-Related Diseases

**DOI:** 10.3390/bios15040232

**Published:** 2025-04-05

**Authors:** Tae-In Park, Ah Hyun Yang, Bashistha Kumar Kanth, Seung Pil Pack

**Affiliations:** 1 Department of Biotechnology and Bioinformatics, Korea University, Sejong 30019, Republic of Korea; jjuft@korea.ac.kr (T.-I.P.); dkgus315@korea.ac.kr (A.H.Y.); 2 Department of Food Science and Nutrition, Dong-A University, Pusan 602760, Republic of Korea; bashistha.kanth@gmail.com

**Keywords:** aptamer, aging, age-related disease, diagnosis, therapeutics, circadian rhythm

## Abstract

In the 21st century, the demographic shift toward an aging population has posed a significant challenge, particularly with respect to age-related diseases, which constitute a major threat to human health. Accordingly, the detection, prevention, and treatment of aging and age-related diseases have become critical issues, and the introduction of novel molecular recognition elements, called aptamers, has been considered. Aptamers, a class of oligonucleotides, can bind to target molecules with high specificity. In addition, aptamers exhibit superior stability, biocompatibility, and applicability, rendering them promising tools for the diagnosis and treatment of human diseases. In this paper, we present a comprehensive overview of aptamers, systematic evolution of ligands by exponential enrichment (SELEX), biomarkers associated with aging, as well as aptamer-based diagnostic and therapeutic platforms. Finally, the limitations associated with predicting and preventing age-related conditions are discussed, along with potential solutions based on advanced technologies and theoretical approaches.

## 1. Introduction

Most life on Earth must inevitably undergo the process of aging triggered by the accumulation of internal and external stress factors, including genetic defects, disease, and environmental influences. In recent decades, many technologies for diagnosis and treatment against various stresses have been developed, contributing to the rapid increase in life expectancy. However, as life expectancy increases, the emergence of an aging society has introduced new social problems, potentially leading to the unprecedented scenario of a super-aged society within a few decades [1]. Therefore, finding sustainable solutions to address the social and economic burdens imposed by a super-aged society has become a critical contemporary issue.

The most significant issue associated with aging is the progressive accumulation of physical defects at molecular, cellular, tissue, and organ levels, leading to an inactive body and unhealthy mind [2,3]. In response, a variety of diagnostic techniques have been developed to monitor the condition of the body, along with anti-aging strategies. Two principal types of biomarkers are employed to predict and address aging: physiological and biochemical. Physiological method includes measurements of heart rate, electrocardiogram, respiratory rate, body temperature, oxygen saturation, and blood pressure [4]. Furthermore, the integration of wearable sensors enables continuous measurement of physiological condition, long-term monitoring, and collective analysis of physical activity capacity [5,6]. Nonetheless, physiological diagnostics have inherent limitations in evaluating internal health parameters. To complement this limitation, biochemical methods have been adopted to monitor molecular-level changes, using biomarkers derived from various biological samples, including sweat, saliva, tears, blood, and urine. For example, the measurement of telomere length, telomerase activity, and telomeric repeat-containing RNA serves as a nucleotide-based biomarker for assessing biological age [7] and may provide valuable insights into the rate of aging [8]. Based on these markers, the strategies to inhibit telomere shortening and to restore lost telomeres through telomerase activation have shown potential in delaying aging and extending lifespan [9,10]. In addition, senescence-associated β-galactosidase (SA-β Gal) is a well-known protein-based biomarker of aging [11]. Several molecular detection methods using fluorescent probes have been developed to target SA-β-Gal activity [12,13,14]. In the cellular environment of β-Gal overexpression, various therapeutic approaches have been devised to selectively eliminate senescent cells. These methods rely on the hydrolysis of β-D-galactose residues to release cytotoxic drugs specifically within senescent cells [15,16,17]. Moreover, anti-aging research has explored diverse strategies, such as the application of embryonic or induced pluripotent stem cells, the activation of adult stem cells, and the use of antioxidants to improve age-related skin deterioration [18,19].

**Figure 1:**
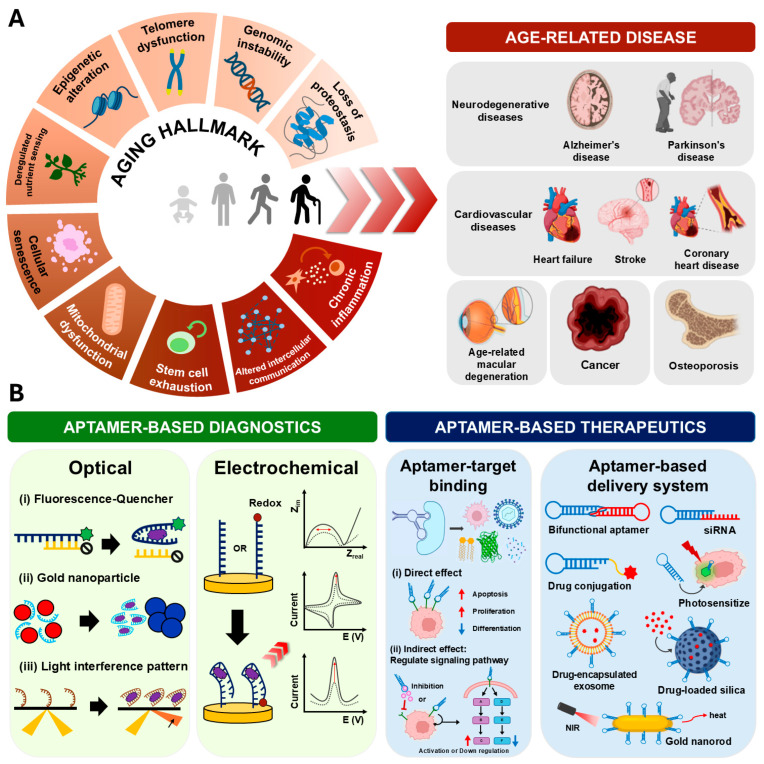
Overview of this study. (**A**) Schematic illustration of aging hallmarks and various age-related diseases. (**B**) Aptamer-based diagnostic and therapeutic application for age-related problems.

Aging is not limited to the accumulation of physical damage; such damage contributes to the development of age-related diseases (Figure 1A), including neurodegenerative diseases [20,21,22], age-related macular degeneration (AMD) [23], cardiovascular diseases (CVD) [24,25,26], cancer [27,28], and others [29,30,31]. These age-related diseases continue to pose significant health risks. The demand for advanced diagnostic and therapeutic tools has grown, prompting the development of innovative technologies [32]. For example, Alzheimer’s disease (AD), a common neurodegenerative disease, is associated with the accumulation of Amyloid-β (Aβ) [33,34]. In preclinical diagnosis of AD, optical and electrochemical biosensors have been developed for detecting Aβ [35,36]. In treatment of AD, Crenezumab, a humanized monoclonal antibody against Aβ discovered by Oskar Adolfsson et al., has demonstrated high affinity for Aβ and the ability to protect cells from the toxicity caused by Aβ oligomers [37]. Multiple clinical trials, including those involving Crenezumab, are currently underway to evaluate treatments for age-related diseases [38,39]. Despite promising developments, some trials have reported inconsistent efficacy, large variations, and side effects due to inhibitory signals on complex cellular pathways or the lack of target specificity. Additionally, biomarkers with multifaceted biological functions in vivo, such as mammalian target of rapamycin (mTOR) and nicotinamide adenine dinucleotide (NAD), may hold the key to overcoming aging-related challenges [40,41], although these also require methods to regulate them appropriately. Therefore, the development of diagnostic tools providing high detection sensitivity, along with therapeutic agents capable of maintaining physiological homeostasis, remains a central objective for advancing treatments for aging and age-related diseases.

The molecular recognition element (MRE) is an important player in the development of advanced biosensors and treatments. Furthermore, their functional efficacy is determined by MRE’s quality, including binding affinity, specificity. Recently, aptamers have emerged as a new MRE to address age-related problems. Aptamers, often called “chemical antibodies”, are short, single-stranded DNA or RNA oligonucleotides that fold into complex three-dimensional structures and bind with high affinity to specific ligands [42]. The principal properties of aptamers are their high specificity and affinity for a wide range of ligands, such as proteins, small molecules, and various types of cells (Table 1) [43,44]. In addition, aptamers can offer several advantages over traditional tools (antibodies, small molecules, peptides), including their small size, high binding affinity, and specificity, low immunogenicity, ease of synthesis, and stability under a range of pH and temperature conditions [45,46,47]. Furthermore, they are cost-effective to produce and can be easily modified for diverse applications [48,49]. These attributes support the growing potential of aptamers for use across numerous fields, including bio-foundry, diagnostics, and therapeutics [50,51].

As the global population continues to age, research on aging-related challenges is expected to be in demand; aptamers are anticipated to play a key role as an MRE diagnostic or therapeutic tool. In particular, aptamer-based diagnostics provide rapid and accurate detection capability for biomarkers through optical and electrochemical biosensors, while aptamer-based therapeutics allow direct or indirect modulation of target cells through aptamer-target binding and aptamer-based delivery systems (Figure 1B). In this review, we present an overview of recent advances in aptamer research concerning aging and age-related diseases, discuss current limitations and potential solutions, and provide future directions for their applications.

## 2. Systematic Evolution of Ligands by Exponential Enrichment (SELEX) for Isolation and Characterization of Aptamer

**Figure 2:**
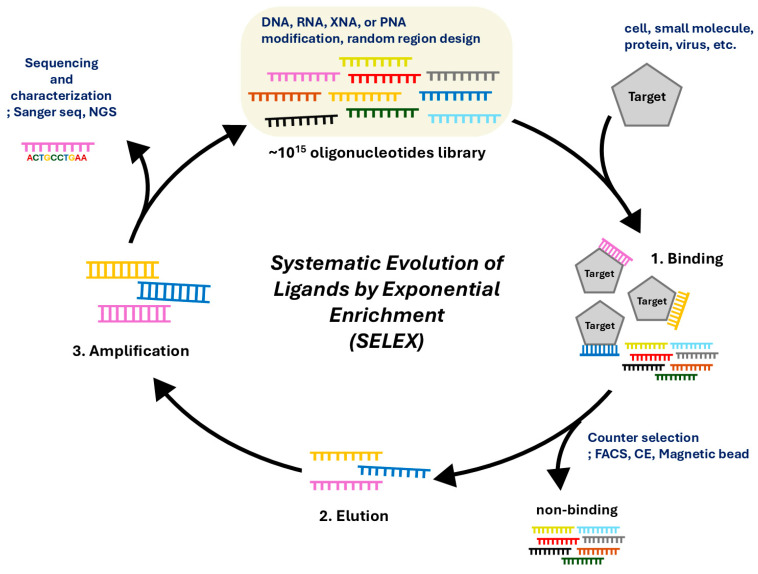
Schematic illustration of Systematic Evolution of Ligands by Exponential Enrichment (SELEX). The SELEX process includes three main steps: binding, elution, and amplification. The cycle is repeated for 1 to 15 rounds, depending on the enrichment strategy. In the last round, aptamers are identified through sequencing and characterization.

Aptamers are typically isolated through Systematic Evolution of Ligands by Exponential Enrichment (SELEX). SELEX, developed by Tuerk and Gold in 1990 [52], consists of iterative cycles with binding (or selection), separation (or elution), and amplification to enrich oligonucleotides that have the desired binding properties for specific targets (Figure 2). The SELEX process begins with a large library pool of random oligonucleotide sequences. The sequences are exposed to a target molecule, separated from non-binding sequences, and subsequently, only target-binding sequences are amplified using polymerase chain reaction (PCR). This cycle is repeated multiple times until target-binding sequences are enriched. Ultimately, the selected sequences are identified through Sanger sequencing or next-generation sequencing (NGS) and are further characterized for their affinity, specificity, and binding sites.

The conventional SELEX process described above typically involves 10 to 20 rounds of selection to discover high-affinity aptamers. However, the conventional SELEX has significant limitations, which include a time-consuming, labor-intensive process and requiring multiple selection cycles to obtain high-affinity aptamers. Furthermore, the absence of real-time monitoring during selection cycles poses a limitation in the assessment of binding efficiency and sequence enrichment. To address these limitations, various advanced SELEX techniques have been developed, incorporating modern technologies. For example, capillary electrophoresis (CE)-SELEX utilizes an electric field to separate molecules based on size and charge, enabling efficient aptamer isolation [53]. Magnetic bead-based SELEX employs magnetic beads immobilized with either oligonucleotides or target molecules to enhance selection efficiency [54]. Fluorescence-activated cell sorting (FACS)-SELEX integrates high-throughput sorting to identify aptamers with desired binding characteristics [55]. This technique enables real-time monitoring of the selection process, ensuring the enrichment of aptamers with high specificity and affinity. Graphene oxide (GO)-SELEX is an immobilization-free method that simplifies the selection process through the absorption of DNA onto GO surfaces [56]. This selective adsorption helps reduce background noise, thereby ensuring the enrichment of strong target-binding aptamers. Finally, Cell-SELEX is a technique that finds aptamers for use in living systems by directly targeting intact cells [57]. These aptamers can recognize specific cell surface markers and are effective in complex biological environments with challenges, including rapid degradation and non-specific interactions.

Each of these advanced SELEX techniques introduces distinct technical advantages, improving aptamer-target affinity while reducing the number of required selection rounds. Importantly, the evolution of SELEX methodologies has significantly contributed to the field of aptamer research, facilitating the development of novel diagnostic and therapeutic agents.

## 3. Biomarkers for Aging and Age-Related Diseases

The selection of suitable targets constitutes an important step in the development of aptamer-based diagnostics and therapeutics. A comprehensive understanding of biomarkers will enhance the potential applications of aptamer. In this section, we explore key biomarkers associated with aging and age-related diseases.

Aging manifests in different parts of the body, with each defect corresponding to distinct biological hallmarks (Figure 1A). According to López-Otín et al., the representative hallmarks of aging can be categorized into primary, antagonistic, and integrative phases [3]. First, the primary hallmark is biological damage at DNA, protein, and organelle levels. These include genomic instability [58], telomere dysfunction [59,60], epigenetic alterations [61,62], loss of proteostasis [63], and disabled macroautophagy [64]. Second, the antagonistic hallmark is a reflective response to damage that has beneficial effects on physical development and maintenance at a young age. However, this response is followed by negative effects, including deregulated nutrient-sensing [65], mitochondrial dysfunction [66], and cellular senescence [67]. Finally, the integrative hallmark is an accumulation of fatal damage raised by primary and antagonistic hallmarks, which includes stem cell exhaustion [68,69], altered intercellular communication [70], chronic inflammation [71], and dysbiosis [72]. Understanding the mechanisms of aging hallmarks is essential to establishing anti-aging strategies.

Furthermore, the accumulation of all these hallmarks contributes not only to aging but also to the increased risk of various age-related diseases, including neurodegenerative diseases, AMD, CVD, osteoporosis, and cancer. Each age-related disease has its own unique biomarkers, which reflect pathological mechanisms and serve as diagnostic indicators and potential targets for treatment (Table 2). Neurodegenerative diseases, including dementia, AD, and Parkinson’s disease (PD), are commonly observed in older individuals or those undergoing the aging process. AD and PD are characterized by progressive degeneration of the nervous system, resulting in cognitive and memory impairment, and motor dysfunction [73]. Although definitive causes have not been fully identified, prior studies have demonstrated that specific proteins, such as amyloid beta (Aβ) [34], tau protein [74], and alpha-synuclein (α-syn) proteins [75], are identified as biomarkers of neurodegenerative diseases. The accumulation of Aβ plaques, tau neurofibrillary tangles, and aggregated α-syn has been shown to cause neuronal damage and death, ultimately disrupting the nervous system and impairing normal brain [76,77]. Additionally, they have shown molecular crosstalk and synergistic interactions in neurodegenerative diseases [78]. Therefore, these neurotoxic can be considered as major biomarkers in the monitoring and treatment of neurodegenerative diseases.

AMD is a progressive retinal disease that primarily affects individuals over the age of 50. Common symptoms of AMD include visual impairment, difficulty recognizing faces, and loss of central vision. In addition, AMD is characterized by the degeneration of photoreceptors in the macula, the central region of the retina [79]. Notably, AMD has been classified into two distinct types: dry (atrophic) and wet (neovascular). The atrophic AMD is marked by progressive thinning of the macula, leading to the accumulation of yellow deposits known as drusen beneath the retina [80]. In contrast, neovascular AMD is characterized by the abnormal growth of blood vessels (neovascularization), a process driven by vascular endothelial growth factor (VEGF). VEGF promotes unregulated neovascularization in the retina, and these neovascularized blood vessels are fragile and prone to leaking fluid or blood, leading to rapid and severe vision loss [81]. Additional biomarkers implicated in the progression of AMD include oxidative stress markers such as malondialdehyde (MDA) and inflammatory cytokines such as interleukin-6 (IL-6) [82]. These biomarkers are associated with chronic inflammation and oxidative damage, which contribute to the deterioration of retinal tissue. Over time, this results in photoreceptor cell death and macular dysfunction [83]. Therefore, assessing VEGF, MDA, and IL-6 levels within the retina represents a promising strategy for the early diagnosis and prevention of AMD. Moreover, the suppression of excessive VEGF, MDA, and IL-6 activation has shown potential as an effective therapeutic approach for the management of AMD.

CVD is a group of diseases affecting the heart and blood vessels, including coronary artery disease, heart failure, and stroke. Common symptoms of CVD include chest pain, shortness of breath, fatigue, and, in some cases, sudden cardiac death. Among the major biomarkers associated with CVD is C-reactive protein (CRP), an inflammatory biomarker [84]. Elevated CRP levels indicate systemic inflammation and stimulate the production of inflammatory cytokines, which contribute to endothelial dysfunction—an early indicator of atherosclerosis [85]. This inflammatory response, driven by CRP, destabilizes arterial plaques and significantly increases the risk of heart attack and stroke. Another clinical biomarker for CVD is cardiac troponin, which is released into the bloodstream during myocardial injury. Troponin contributes to the progression of CVD by signaling ongoing myocardial stress and damage, while triggering additional inflammatory and fibrotic responses in heart tissue. Therefore, regular monitoring of CRP and troponin levels is considered an effective means of predicting the progression of CVD and guiding clinical decisions regarding patient management [86]. In another biomarker, inhibiting the activity of integrin avβ3, an angiogenesis-related factor, and von Willebrand factor (vWF), a platelet aggregation protein, will be possible to improve vascular occlusion and normalize blood flow, potentially providing useful therapeutic avenues for the treatment of CVD [87,88].

Osteoporosis is commonly induced by hormonal changes or aging and is characterized by the loss of bone mass and deterioration of bone tissues, resulting in pain, reduced height, and an increased risk of fracture. Most biomarkers associated with osteoporosis are closely related to the bone remodeling process, including bone formation (osteogenesis), bone resorption (osteoclastogenesis), and bone turnover regulations [89]. Osteogenesis is a complex biological process influenced by various biomarkers, such as osteocalcin (OC), procollagen type 1 N-terminal propeptide (P1NP), and procollagen type 1 C-terminal propeptide (P1CP). P1NP, a byproduct of type I collagen synthesis, serves as an indicator of osteoblastic activity and declines with age due to decreased osteoblast function and impaired Wnt/β-catenin signaling. In contrast, a key biomarker of osteoclastogenesis is carboxy-terminal cross-linking telopeptide of type I collagen (CTX-1). CTX-1 is a vital indicator of osteoclast-mediated degradation of type I collagen, which increases with age-related oxidative stress, enhancing NF-κB (RANK) and NF-κB ligand (RANKL) signaling pathways [90]. RANKL plays a crucial role in regulating bone turnover, as do advanced glycation end products (AGEs) and sclerostin. Sclerostin, a Wnt signaling inhibitor, increases with aging, thereby suppressing osteoblast and bone formation. Furthermore, AGEs accumulate in bone collagen over time, impairing its mechanical properties and promoting inflammation, which exacerbates bone turnover imbalance. Collectively, these biomarkers significantly contribute to the development of age-related osteoporosis by affecting major signaling pathways, including RANK/RANKL/OPG [91], Wnt/β-catenin, and oxidative stress. Appropriate modulation of these pathways will provide significant benefits in the early diagnosis and treatment of osteoporosis.

Cancer is defined by uncontrolled cell proliferation and the ability to invade adjacent tissues or metastasize to distant organs through the bloodstream. Cancer can arise in various vital organs, including the thyroid, lungs, breast, prostate, intestines, and skin. In addition, these are often accompanied by symptoms such as weight loss, fatigue, tissue death, skin necrosis, or impaired bodily function. Cancer is closely associated with aging, as it contributes to the accumulation of genetic mutations, epigenetic changes, and cellular senescence, increasing susceptibility to malignant transformation. For example, the accumulation of senescence-associated secretory phenotype (SASP) is a hallmark of aging that creates a tumor-promoting environment by releasing inflammatory cytokines and growth factors that disrupt tissue homeostasis and stimulate cancer progression [92]. Furthermore, SASP components, such as IL-6 [93], have been shown to activate the STAT3 signaling pathway, which supports cancer cell proliferation and resistance to apoptosis. Another key biomarker is p16^INK4a^, a regulator of cellular senescence that inhibits cyclin-dependent kinases and induces cell cycle arrest [94]. These biomarkers influence the PI3K/AKT/mTOR pathway and enhance cell survival and growth, and the Wnt/β-catenin pathway, which regulates cell proliferation and differentiation [95]. Increased p16^INK4a^ expression may contribute to oncogenic environment transition by disrupting cell cycle control mechanisms. Moreover, additional biomarkers, including tumor-derived exosomes, immune checkpoint molecules such as programmed cell death ligand 1 (PD-L1) [96], and aging-associated microRNA (e.g., miR-146a, miR-21) [97], have been effective in the diagnosis or treatment of cancer patients.

In the modern era of extended human longevity, the preparation for aging and age-related diseases has become a pivotal challenge. In addition, these issues can reduce individuals’ physical capacity and quality of life, potentially diminishing the size of the economically active population. Thus, the societal burden associated with aging is projected to increase substantially. To mitigate these effects, it is essential to identify and address the hallmarks of aging and biomarkers associated with age-related diseases by incorporating novel, reliable, and effective molecular recognition tools, such as aptamers.

**Table 2:** The biomarkers for age-related diseases.

Diseases	Biomarkers	Function	Ref.
Neurodegenerative diseases	Amyloid beta (Aβ)	Formation of amyloid plaques; toxic to nerve cells	[33]
	Tau protein	Neurofibrillary tangles; toxic to nerve cells	[77]
	Alpha-synuclein (α-syn)	Aggregates in Lewy bodies; decreases resistance to neuronal apoptosis	[98]
Age-related macular degeneration (AMD)	Vascular endothelial growth factor (VEGF)	Induces abnormal blood vessel growth beneath the retina	[99]
	Malondialdehyde (MDA)	Promote cytotoxicity and VEGF expression in retinal tissue	[100]
	Interleukin-6 (IL-6)	Pro-inflammatory cytokines; induces VEGF expression and choroidal neovascularization	[101]
Cardiovascular disease (CVD)	C-reactive protein (CRP)	Inflammatory-associated biomarker; promotes endothelial dysfunction	[84]
	Von Willebrand Factor (vWF)	Promotes platelet adhesion and clot formation; increases the risk of atherosclerosis	[88]
	Integrin avβ3	Cell surface receptor; mediates angiogenesis, and endothelial cell adhesion	[102]
Osteoporosis	Osteocalcin (OC)	Indicators of bone formation (osteogenesis)	[103]
	Procollagen type 1 N-terminal propeptide (P1NP)		[104]
	Procollagen type 1 C-terminal propeptide (P1CP)		[105]
	Carboxy-terminal cross-linking telopeptide of type I collagen (CTX-1)	Indicators of bone resorption (osteoclastogenesis)	[106]
	NF-κB ligand (RANKL)	Regulators of bone turnover	[90]
	Advanced glycation end products (AGEs)		[107]
	Sclerostin		[108]
Cancer	Senescence-associated secretory phenotype (SASP)	Promotes proliferation and metastasis of cancer; induces inflammatory cytokine release	[51]
	p16^INK4a^	Tumor suppressor; inhibits cyclin-dependent kinase CDK4	[109]
	Programmed cell death ligand 1 (PD-L1)	Suppresses adaptive immune responses by binding to PD-1	[110]
	Tyrosine-protein kinase-like 7 (PTK7)	Transduces extracellular signals across the cell membrane	[111]

## 4. Aptamer-Based Diagnosis and Therapeutics for Aging and Age-Related Diseases

Aptamers are under active investigation for a wide range of applications, including diagnostics, drug delivery, and therapeutics. This is also employed in biosensing platforms for detecting various targets, including pathogens and toxins. The incorporation of aptamers into biosensing systems enables real-time, cost-effective, and non-invasive analysis of biological samples, supporting early diagnosis and improved disease management. Furthermore, aptamers have emerged as promising tools for the treatment of aging and age-related diseases by binding selectively to disease-associated proteins and modulating pathological signaling pathways [112,113,114]. For example, aptamers targeting advanced glycation end products (AGEs) and their receptor (RAGE) have shown potential in alleviating complications caused by aberrant protein modifications, which are implicated in disorders such as AD, osteoporosis, and CVD [115]. This section provides comprehensive information on precision diagnostics and therapeutics tools aimed at delaying or mitigating age-related diseases such as neurodegenerative diseases, CVD, AMD, osteoporosis, and cancer.

### 4.1. Aptamer-Based Diagnosis

The high sensitivity and stability of aptamers toward their targets in aptamer-based diagnostic approaches allow them to be utilized as powerful tools for detecting and diagnosing age-related diseases. Upon binding to target molecules, the aptamer generates measurable output signals that can be captured by various aptamer-based biosensors, including optical and electrochemical sensing platforms (Table 3 and Table 4).

#### 4.1.1. Optical Sensing

Optical sensing in aptamer-based biosensors involves the detection of target molecules through changes in optical signals, including fluorescence, luminescence, colorimetry, surface plasmon resonance (SPR), and biolayer interferometry (BLI) [134,135]. First, fluorescence-based aptasensors typically utilize a fluorophore and a quencher conjugated to, or placed near, the aptamer to monitor target binding. Subsequently, the aptamer-target binding induces a structural shift in the aptamer and increases the distance between the fluorophore and quencher, resulting in a detectable fluorescence signal. For example, Le Minh Tu Phan et al. developed a nitrogen-doped carbon dot (NCD)-based aptasensor for the detection of tau protein, a key AD biomarker [116]. In this system, NCD fluorescence was initially quenched by the aptamer. Upon binding with the tau protein, the aptamer was released from the NCD surface, restoring fluorescence and enabling signal detection. This aptasensor achieved a limit of detection (LOD) of 3.64 ng/mL, demonstrating significant potential for early diagnosis of AD. Next, luminescence-based detection has various variations, of which chemiluminescence (CL)-based detection is the most commonly applied in age-related disease biosensors. CL-based aptasensors operate by emitting light through a chemical reaction. For example, Siwen Shan et al. developed an aptamer sandwich-based CL assay for VEGF165 detection [117]. Two VEGF binding aptamers produced light with the hydrolysis of 4-methoxy-4-(3-phosphatephenyl)-spiro-(1,2-dioxetane-3,2-adamantane) (AMPPD) under the catalysis of alkaline phosphatase (ALP). This CL-based aptasensor exhibited an LOD of 1 ng/mL and demonstrated high accuracy in VEGF165 quantification.

**Figure 3:**
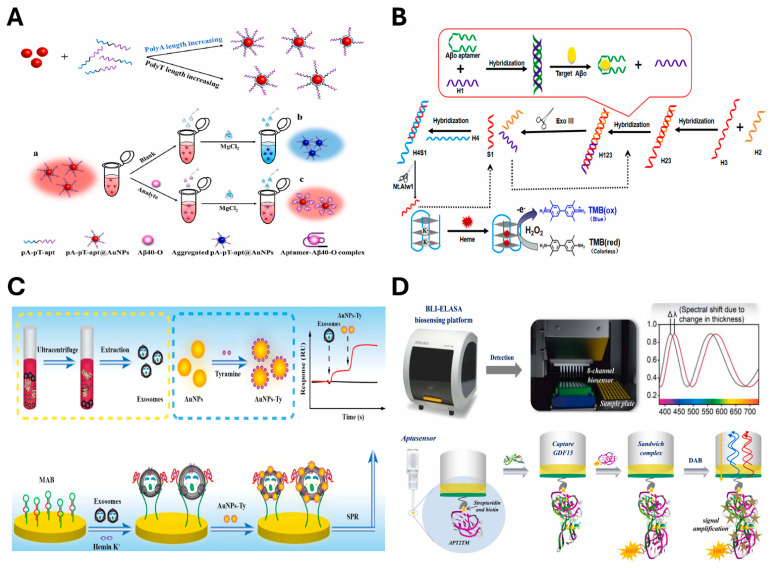
Aptamer-based optical biosensing platforms. (**A**) Label-free colorimetric aptasensor based on an aptamer-polythymine (polyT)-polyadenine (polyA)- gold nanoparticle (pA-pT-apt@AuNPs) platform for Aβ40-O detection. Reprinted with permission from [118]. (**B**) Schematic illustration of a dually amplified colorimetric aptasensor for Aβo detection. Reprinted with permission from [119]. (**C**) Schematic illustration of SPR aptasensor for HER2-positive exosomes using tyramine signal amplification triggered by target-induced molecular aptamer beacon conversion. Adapted from [122]. (**D**) Schematic illustration of novel BLI-ELASA-based aptasensor for GDF15 detection. Adapted with permission from [124].

Colorimetric assays are based on the color changes triggered by the interaction between aptamers, target molecules, and nano-materials such as gold nanoparticles (AuNPs). These assays are advantageous for their simplicity and rapid visual readouts. For instance, Ying Tu et al. developed a label-free colorimetric aptasensor based on aptamer-polythymine (polyT)-polyadenine (polyA)-gold nanoparticles (pA-pT-apt@AuNPs) for amyloid-β1-40 oligomers (Aβ40-O) detection (Figure 3A) [118]. In this design, the polyA segment was immobilized on the AuNP surface, whereas the aptamer specifically recognized Aβ40-O. In the absence of a target, the aggregation of pA-pT-apt@AuNPs could be induced by MgCl_2_, resulting in a color shift from red to blue. When a target was present, the aptamer folded upon recognition, forming the aptamer-Aβ40-O complex on the surface of the pA-pT-apt@AuNPs conjugate. This complex effectively stabilized the colloidal particles against salt-induced aggregation, maintaining the red color and achieving an LOD of 3.03 nM for Aβ40-O. Furthermore, Miao Chen et al. developed an aptamer-based amyloid-β oligomer (Aβo) sensor that exhibits a dual-amplification colorimetric signal (Figure 3B) [119]. This sensor utilized an Aβo-specific aptamer as the recognition element and a hemin/G-quadruplex DNAzyme for signal transduction. Signal enhancement was achieved through cyclic amplification mechanisms involving exonuclease III (Exo III) and nicking enzyme (Nt.Alw1). Hybridization of the AβO aptamer with the H1 oligonucleotide initiated a series of cyclic amplification reactions, resulting in an LOD of 0.23 pM. Other widely adopted colorimetric strategies include enzyme-linked immunosorbent assays (ELISA) and enzyme-linked oligonucleotide assay (ELONA), also referred to as the enzyme-linked aptamer assay (ELAA) or enzyme-linked aptamer sorbent assay (ELASA) [124]. ELASA is widely utilized in point-of-care (POC) diagnostics due to its simplicity, rapid processing time, and minimal procedural complexity.

More recently, advanced technologies such as SPR and BLI have been integrated with aptamers to develop ultra-sensitive biosensors. SPR-based detection is a technique that provides a label-free and real-time method for monitoring targets by measuring changes in the refractive index near the sensor surface when aptamers bind to target molecules. This approach enables direct detection and quantification of biomolecular interactions in real-time. In SPR-based aptasensors, the biomarker of CVD could also be detected by a 3′-thiol-modified 6th-62-40 aptamer binding against CRP, exhibiting an LOD from 10 pM to 1 nM [121]. Additionally, Wenqin Chen et al. reported the detection of human epidermal growth factor receptor 2 (HER2)-positive exosomes using a molecular beacon that combined G-quadruplex (G4) DNA and a HER2 aptamer (Figure 3C) [122]. In this system, the G4 DNA-HER2 aptamer was immobilized on the sensor chip to capture HER2-positive exosomes. The exposed G4 DNA formed G4-hemin complexes that exhibited peroxidase-like activity, catalyzing the deposition of tyramine-coated gold nanoparticles (AuNPs-Ty) on the exosome membrane. This resulted in an enhanced SPR signal and enabled detection over a wide linear range from 1.0 × 10^4^ to 1.0 × 10^7^ particles/mL. BLI is another optical technique that monitors the changes in the interference pattern of reflected light from the biosensor surface, allowing real-time measurement of biomolecular interactions. BLI-based aptasensors operate by immobilizing a probe or analyte on the sensor tip and require only a small sample volume. This technique also allows for accurate measurement of binding kinetics and affinity between aptamer and their target. For example, Iva Ziu et al. reported the BLI-based label-free aptasensor for tau441 protein detection, exhibiting an LOD of 6.7 nM [123].

Moreover, biosensors with high sensitivity to target molecules have been developed by integrating multiple optical sensing techniques. Shunxiang Gao et al. introduced the BLI-ELASA platform, which combines the high sensitivity of BLI with the amplification efficiency of ELASA (Figure 3D) [124]. This platform utilized APT2TM aptamers to detect growth differentiation factor-15 (GDF15), a biomarker of glaucoma and potentially aging, and achieved an exceptionally low LOD of 5–6 pg/mL. In addition, Francesca Torrini et al. reported a hybrid platform that integrated SPR and ELONA assays. This system used two different aptamers in a sandwich format to detect recombinant human cardiac troponin T isoform 6 (cTnT3), a key cardiac biomarker. The SPR-ELONA method demonstrated high sensitivity, achieving LODs of 3.42 nM with the direct method and 3.13 nM with the sandwich method [125]. Collectively, these aptamer-based optical sensing technologies provide effective detection of biomarkers associated with age-related diseases. Their high sensitivity and real-time monitoring capabilities position them as promising candidates for integration into POC diagnostics.

#### 4.1.2. Electrochemical Sensing

Aptamer-based electrochemical biosensor is a device that uses a sensing electrode surface immobilized with aptamers and detects targets through changes in the three-dimensional structure of the aptamer [136]. This structural alteration can be transduced into an electrical signal and output on the device interface. These biosensors offer advantages such as rapid response, high sensitivity, and selectivity, making them highly suitable for the diagnosis of age-related diseases [137,138].

Electrochemical aptasensors are classified based on their signal output mechanisms, including voltametric, amperometric, impedance, and potentiometric [139]. In voltammetric and amperometric detection modes, including cyclic voltammetry (CV), square wave voltammetry (SWV), differential pulse voltammetry (DPV), and pulsed amperometric detection (PAD), the current flowing at the electrode can be altered by aptamer–target interactions. In addition, the use of electrochemically active labels conjugated to the aptamer termini can amplify the output signal through redox reactions. First, CV-based aptasensors represent a fundamental electrochemical detection strategy. These sensors measure current variations that result from the movement of electrons in response to aptamer-target binding. The induced potential change leads to a measurable current shift, allowing accurate target detection. For instance, Xian-Ming Fu et al. reported a CV-based aptasensor using DNA-templated Ag/Pt bimetallic nanoclusters [128]. This system incorporated amino-Apt13 and template-Apt12 in a label-free, sandwich-type configuration for VEGF detection, achieving an LOD of 4.6 pmol/L.

**Figure 4:**
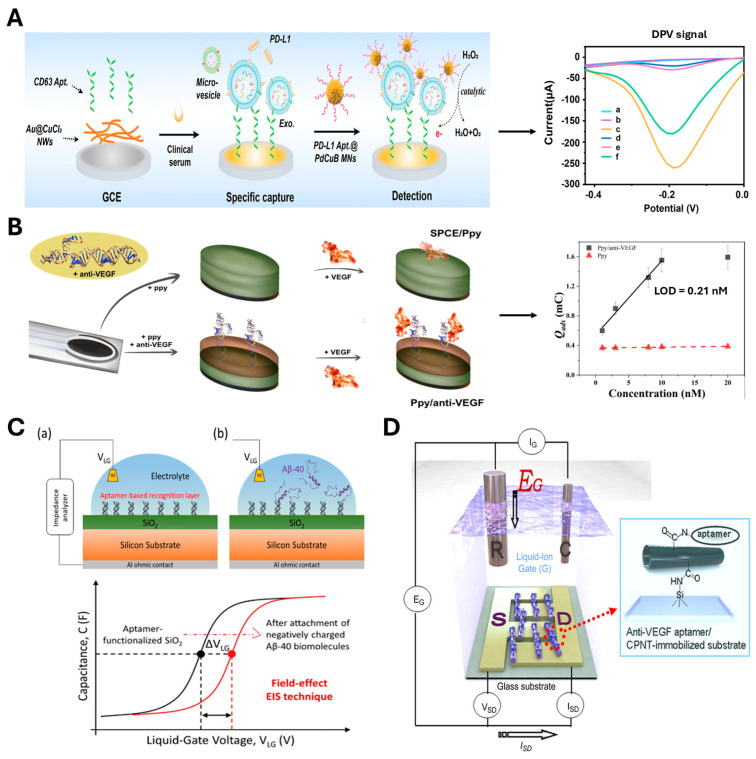
Aptamer-based electrochemical biosensors. (**A**) Schematic illustration of a sandwich-type, non-invasive electrochemical aptasensor for PD-L1+ exosome detection. DPV signals are generated by the peroxidase-like catalytic activity of PdCuB MNs. Adapted from [131]. (**B**) Schematic illustration of PAD-based aptasensor. Polypyrrole (Ppy) immobilizes anti-VEGF aptamers on the screen-printed carbon electrode (SPCE) surface for PAD evaluation via potential pulse sequences at +0.6 V and +0 V. Q_ads_ is the changes in charge due to adsorbed species. Adapted with permission from [132]. (**C**) Schematic illustration of EIS-based aptasensor functionalized with Aβ-40-specific aptamers, showing surface potential detection before (**a**) and after (**b**) Aβ-40 peptide interaction. Adapted with permission from [129]. (**D**) Schematic illustration of FET-based reusable aptasensor using anti-VEGF aptamer-conjugated carboxylated polypyrrole nanotubes (CPNTs), which detect the signal through a three-terminal transistor configuration. Reprinted with permission from [133].

In voltammetric detection, Mingjian Lang et al. developed an ultrasensitive biosensor for the detection of cardiac troponin I (cTnI) based on terminal deoxynucleotidyl transferase-mediated signal amplification and recognition between cTnI and the aptamer [140]. The biosensor utilized a gold electrode modified with a probe and a methylene blue-polyA hybridized with extended polyT to amplify the electrochemical signal. This system achieved an LOD of 40 pg/mL, as determined by SWV analysis. In addition, Sofia G. Meirinho et al. reported a label-free electrochemical aptasensor to detect recombinant human osteopontin (rhOPN), a relevant breast cancer biomarker, exhibiting an LOD of 1.3 ± 0.1 nM by using both SWV and CV [127]. In DPV-based aptasensor, Luyue Chang et al. designed a sandwich electrochemical aptasensor for detecting PD-L1-positive (PD-L1+) exosomes—potential biomarkers for diagnosing non-small cell lung cancer (Figure 4A) [131]. The CD63 aptamer was immobilized on an electrode modified with Au@CuCl_2_ nanowires to capture the exosomes. Additionally, ternary metal-metalloid palladium-copper-boron alloy microporous nanospheres (PdCuB MNs), modified with a PD-L1 aptamer and exhibiting peroxidase-like activity, were used to enhance signal amplification. This sensor achieved an LOD of 36 particles/mL and holds promise for rapid, non-invasive, and accurate on-site cancer diagnosis using sandwich electrochemical strategies.

In amperometric detection, PAD is an electrochemical method that measures current responses to a sequence of potential pulses applied to the electrode surface. PAD is often integrated with aptamers to monitor redox reactions or electron transfer events that occur during aptamer–target interaction. This biosensor may also use labels such as enzymes, methylene blue, or ferrocene to amplify the output signal and improve sensitivity, providing insights into the kinetics and mechanisms of binding processes. For example, Viktorija Liustrovaite et al. developed a PAD-based aptasensor using a screen-printed carbon electrode (SPCE) modified with polymerized polypyrrole (Ppy) (Figure 4B) [132]. Three self-assembling DNA aptamers, including combSl2B, stalkGTG, and r_stalkGTG, were immobilized on the Ppy layer on the SPCE surface to capture VEGF, achieving an LOD of 0.21 nM. Collectively, these aptasensors based on voltammetric and amperometric detection methods have shown significant potential for *in vitro* applications in early cancer diagnosis and treatment monitoring, allowing rapid and efficient screening of patient samples.

In impedance-based biosensors, electrochemical impedance spectroscopy (EIS), the interaction between the aptamer and the target induces measurable changes in the conductivity and capacitance at the electrode surface. The interfacial property changes of the electrodes, including resistance and capacitance, can be used to detect molecular interactions with high sensitivity and specificity. For example, Yurii Kutovyi et al. reported an EIS-based sensor incorporating a thin SiO_2_ dielectric layer functionalized with Aβ-40-specific aptamers for detecting Aβ-40 peptides (Figure 4C) [129]. This aptasensor could successfully detect ultra-low concentrations of the target, by measuring surface potential changes across various target concentrations, ranging from 0.1 pg/mL to 10 μg/mL.

Finally, potentiometric aptasensors, such as field-effect transistor (FET)-based aptasensors, employ a semiconductor channel (e.g., silicon or graphene) integrated with source, drain, and gate electrodes. This aptasensor detects subtle changes in conductivity and current flows caused by electric field shifts in response to the binding of analytes to aptamers at the semiconductor surface. The unique properties of semiconductors allow real-time and highly specific detection, making aptasensors suitable for miniaturization and high-throughput screening. Oh Seok Kwon et al. developed a high-performance FET-based sensor using an anti-VEGF RNA aptamer (CPNT2-aptamer) conjugated to carboxylated polypyrrole nanotubes (CPNTs) for detection of VEGF, a known cancer biomarker (Figure 4D) [133]. This aptasensor was designed with a three-terminal transistor comprised of drain, source, and gate electrodes; the anti-VEGF aptamer-conjugated CPNTs were immobilized on a glass substrate with drain electrodes in a liquid-ion gate to allow reusable detection of the target. Furthermore, the surface-immobilized CPNT2 aptamer provided VEGF targeting function, whereas the CPNTs facilitated signal amplification, resulting in an LOD of 400 fM. In a separate study, a highly sensitive aptasensor using a silicon (Si) two-layer (TL) nanowire (NW) FET structure was developed and exhibited an LOD of 20 fM against Aβ-40 [129].

Overall, aptamer-based electrochemical biosensors hold significant promise for improving sensitivity, specificity, and versatility in the detection and analysis of biomarkers associated with age-related diseases. Many age-related diseases, including neurodegenerative diseases, AMD, CVD, osteoporosis, and cancer, are well known to involve molecular changes in their unique biomarkers.

From this perspective, electrochemical aptasensors offer substantial potential for early diagnosis and continuous monitoring of these conditions. Moreover, their integration with communication systems and wearable technologies may enable real-time monitoring of age-related biological processes, representing a transformative advancement in contemporary medical diagnostics.

### 4.2. Aptamer-Based Therapeutics

Aptamers serve not only as diagnostic tools for aging and age-related diseases but also exhibit considerable therapeutic potential. Notably, aptamer-based drugs such as pegaptanib and avacincaptad pegol have been approved by the US Food and Drug Administration (FDA) for the treatment of AMD [141,142]. Additionally, aptamers can be chemically modified with various molecules to act as therapeutic agents or delivery vehicles, enabling targeted drug delivery. This section presents several examples of aptamers used either as direct inhibitors or as delivery systems targeting mechanisms of aging and age-related diseases (Table 5).

#### 4.2.1. Anti-Aging Strategy

Aging is a complex phenomenon caused by the accumulation of multiple hallmarks. For this reason, overcoming the fundamental causes of aging remains challenging. However, various methods such as reactive oxygen species (ROS) elimination, protein deacetylation, and anti-inflammatory strategies have been proposed in recent years, and aptamer-based anti-aging approaches have been developed accordingly. First, ROS are primarily generated by mitochondria, and mitochondrial dysfunction leads to excessive ROS production. Increasing levels of ROS induce oxidative stress, which causes extensive damage to cells, organelle membranes, DNA, lipids, and proteins, contributing significantly to the aging process [175]. To reduce such ROS, vitamin C, known for its electron-donating properties, can quickly eliminate ROS. However, vitamin C is not very efficient due to rapid oxidation under environmental factors, including oxygen, pH, temperature, and UV light. According to Sooho Choi et al., Aptamin C can bind to vitamin C and preserve the reduced form of vitamin C. In addition, by delaying the oxidation of vitamin C, Aptamin C can maximize its antioxidant capacity, showing clinical efficacy in the treatment of skin aging [143]. Furthermore, Aptamin C prevents ROS-induced microvascular damage in the brain and protects the blood–brain barrier, demonstrating its therapeutic potential in vascular aging [176]. Second, another anti-aging target is Sirtuin 1, an NAD+-dependent deacetylase, involved in gene regulation, metabolism, DNA repair, and the regulation of cellular senescence [114]. In particular, the repair ability of DNA is opposed to ROS attack on mitochondrial DNA, suggesting that activation of sirtuin 1 is crucial to anti-aging strategies. Rana Faris Salman et al. showed that a Sirtuin 1-specific aptamer can protect keratinocytes from oxidative damage and inhibit skin aging [144]. Third, dysregulated inflammation, often characterized as a cytokine storm, contributes to age-related problems such as immune and cellular senescence [177]. Tumor necrosis factor-alpha (TNF-α) is a key pro-inflammatory cytokine in this process. In addition, TNF-α-targeting aptamers can reduce the side effects associated with inflammation, maintaining the balance between cell death and proliferation. [178,179]. Moreover, SASP, including SA-β-gal, is directly related to cellular senescence, and the localization ability of the aptamer can be employed to selectively eliminate aged cells. For example, the aptamer yly12 binds to the cell adhesion molecule L1 and can be conjugated to prodrugs such as EF24 and hydrogen sulfide (H_2_S), resulting in their accumulation inside senescent cells and finally cell apoptosis [145,146]. Therefore, various strategies using aptamers have great anti-aging potential, including approaches that target key cellular and molecular mechanisms associated with senescence and tissue degeneration.

#### 4.2.2. Treatment of Neurodegenerative Diseases

**Figure 5:**
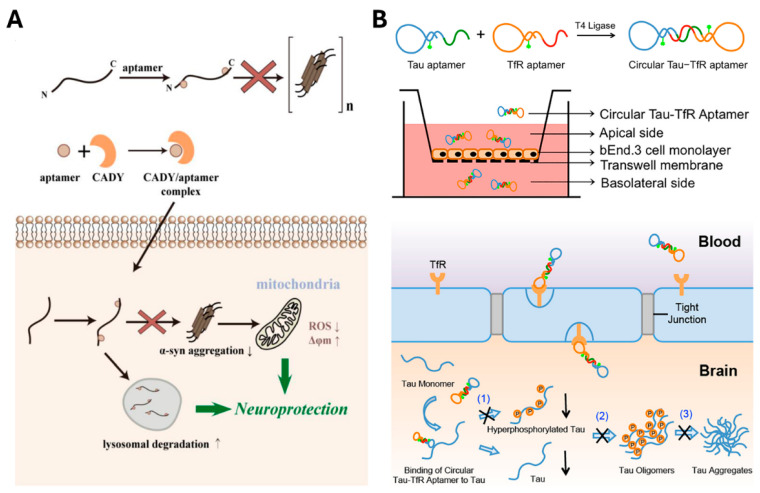
Aptamer-based therapeutic strategies for neurodegenerative diseases. (**A**) Schematic illustration showing high-affinity aptamers binding to the N- and C-termini of α-synuclein, thereby inhibiting its aggregation *in vitro*. Upon cellular uptake, these aptamers reduce α-syn aggregation *in vivo* and facilitate their degradation via the lysosomal pathway, rescuing mitochondrial dysfunction and cellular defects induced by α-syn overexpression. Reprinted from [149]. (**B**) Schematic illustration of a circular bispecific aptamer traversing an *in vitro* BBB model. The aptamer incorporates transferrin receptor (TfR)- and tau-targeting sequences (IT2a) and is labeled with a fluorescein isothiocyanate fluorescence tag. The therapeutic mechanism includes inhibition of (1) tau hyperphosphorylation, (2) tau oligomerization, and (3) tau aggregation. Adapted with permission from [151]. Copyright (2020) American Chemical Society.

As previously discussed, aging affects various regions of the body, particularly the brain, thereby increasing the risk of neurodegenerative diseases, including AD and PD. AD is caused by the accumulation and aggregation of Aβ and tau protein in the brain, while PD is associated with the aggregation of α-syn protein. Therefore, inhibiting the aggregation of these pathological proteins may significantly reduce disease progression. In alignment with this therapeutic concept, aptamers targeting Aβ [147], tau protein [148], and α-syn protein [149] (Figure 5A) have been identified and shown to be effective in protecting neurons under neurodegenerative stress and in reducing neuropathological impairments.

However, a major obstacle to the treatment of neurodegenerative diseases is the blood–brain barrier (BBB), which limits the delivery of therapeutic agents to the central nervous system. The BBB’s selective permeability hinders the effective transport of drugs into the brain, complicating the development of efficient treatments. To address this challenge, various aptamer delivery systems have been engineered, including transferrin receptor (TfR)-targeting aptamers capable of BBB penetration [180], BBB shuttle aptamers [181], and exosome-based delivery systems incorporating neuron-specific peptides [182]. Using this, Xiaowei Li et al. established a tau-TfR bifunctional aptamer with BBB permeability, which was capable of reducing traumatic brain injury-induced cognitive and memory deficits while maintaining strong binding to tau protein *in vitro* and *in vivo* (Figure 5B) [151]. Furthermore, Xiaoxi Ren et al. developed an α-syn targeting aptamer, F5R2, which was packaged within exosomes engineered to include neuron-specific peptides-included exosome. This delivery system successfully transported F5R2 into the brain, where it reduced α-syn aggregation and improved motor impairment [150]. These findings emphasize the potential of aptamers in the treatment of neurodegenerative diseases, as well as the capacity to overcome the restrictive properties of the BBB through various delivery strategies.

#### 4.2.3. Treatment of Age-Related Macular Degeneration

AMD is characterized by abnormal blood vessel growth in the retina. Most treatments focus on the inhibition of angiogenic proteins, such as VEGF. After the discovery of aptamers for VEGF by Jellinek et al. in 1994 [183], Ruckman et al. identified a 2′-F-pyrimidine RNA aptamer that effectively inhibited VEGF binding and significantly reduced VEGF-induced vascular permeability in the skin [184]. subsequently, David R. Guyer developed the pegylated anti-VEGF aptamer EYE001, which not only completely prevented VEGF-mediated vascular leakage but also inhibited neovascularization [152]. Additionally, the first aptamer drug, pegaptanib, was approved by the FDA for AMD patients in 2004 [141]. Other aptamer-based anti-VEGF therapies, including ranibizumab and bevacizumab, have also been developed and shown dramatic visual recovery in AMD patients [185,186,187]. However, some patients had incomplete responses to these therapies, and in severe cases, recurrence of AMD results in vision loss [188,189,190,191]. Consequently, the identification of additional therapeutic targets has become a priority. Platelet-derived growth factor (PDGF) and fibroblast growth factor 2 (FGF2) have emerged as promising targets for the treatment of AMD. The PDGF-targeting aptamer E10030 has been proven to reduce neovascularization by 85% [153,154]. Similarly, the FGF2-targeting aptamer RBM-007 effectively inhibits neovascularization and subretinal fibrosis and possesses a twofold longer half-life than other anti-VEGF agents [155]. When co-administered with ranibizumab, RBM-007 provided a synergistic effect in suppressing neovascularization. Collectively, these findings suggest that combined administration of anti-VEGF, anti-PDGF, and anti-FGF2 aptamers can be a pivotal therapeutic strategy for treating AMD.

#### 4.2.4. Treatment of Cardiovascular Diseases

CVD results from impaired or obstructed blood flow; they typically begin with endothelial injury, followed by chronic inflammation and thrombosis [192]. In particular, phenotypic changes in vascular smooth muscle cells (VSMCs) associated with aging can trigger proliferation and migration, leading to vascular stiffening, calcification, aneurysm formation, and rupture. Hong-Bing Wu et al. reported that an integrin avβ3-aptamer inhibited Ras-phosphatidylinositol-4,5-bisphosphate 3-kinase/mitogen-activated protein kinase (PI3K/MAPK) signaling, thereby preventing VSMC proliferation and migration [156]. Additionally, Paloma H. Giangrande et al. demonstrated that Apt14, a VSMC-specific aptamer, discovered through Cell-SELEX [193], blocked VSMC migration by activating platelet-derived growth factor receptor (PDGFR)-β and inhibiting phosphatidylinositol 3-kinase/protein kinase-B (PI3K/Akt) signaling [157]. Apt14 was also delivered directly to the arterial medial layer using an occlusion perfusion catheter, which enhanced aptamer retention within the vessel wall and suggested its potential as a safer and more effective treatment [194].

Thrombosis resulting from platelet aggregation poses a serious risk for CVD progression [192]. The most efficient way to prevent thrombosis involves disrupting the vWF bridge between exposed collagen and platelet glycoprotein GPIb. Accordingly, various vWF aptamers have been discovered, and their anti-vWF activity inhibits platelet aggregation and promotes vascular recanalization in thrombotic occlusions [158,159,160,161,195]. Additionally, aging can trigger autoimmune diseases, and autoimmune-induced tissue destruction can lead to a variety of diseases. In particular, autoantibodies against G-protein-coupled receptors (GPCR-AABs) may induce serious cardiovascular conditions [196]. Gerd Wallukat et al. developed an aptamer that neutralizes GPCR-AABs, demonstrating its therapeutic potential in treating cardiomyopathy caused by such autoimmune activity [162]. As a result, aptamer-based approaches offer versatile, safe, and effective options for CVD treatment.

#### 4.2.5. Treatment of Osteoporosis

The primary pharmacological treatment for osteoporosis involves bisphosphonates, which are characterized by excessive osteoclast-mediated bone resorption. However, long-term bisphosphonate use has been associated with adverse effects such as osteonecrosis, microfractures, and skeletal fragility [197]. Therefore, new therapeutic strategies that minimize side effects while maintaining efficacy are needed. In this context, aptamer-based therapies have emerged as promising alternatives, offering high biocompatibility.

**Figure 6:**
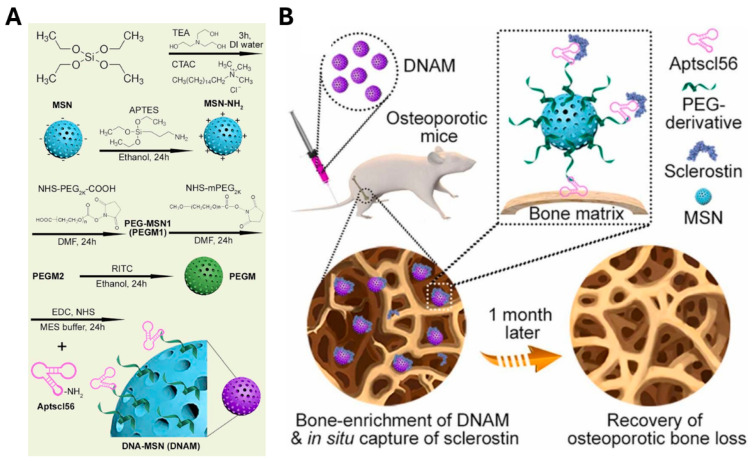
Aptamer-based therapeutic strategies for osteoporosis using anti-sclerostin DNA aptamer. (**A**) Schematic illustration of MSN fabrication and the synthesis steps of DNA-nanomedicine (DNAM) using surface-modified dendritic mesoporous silica nanoparticle (MSN) with anti-sclerostin DNA aptamers (Aptscl56) after amination (MSN-NH_2_) and PEGylation (PEGM). (**B**) Schematic illustration of the mechanism of the bone-targeting and therapeutic efficacy of sclerostin aptamers. DNAM attaches to bone calcium and regulates serum sclerostin level, enabling osteoporosis treatment. (**A**,**B**) are adapted with permission from [165].

Aptamers are often employed as delivery agents for siRNAs and antagomicroRNA (antagomiR) in osteoporosis treatment. For instance, osteoblast aptamer (CH6)-functionalized osteogenic pleckstrin homology domain-containing family O member 1 (Plekho1) siRNA-encapsulated lipid nanoparticles have been shown to promote bone formation, improve bone microarchitecture, increase bone mass, and enhance mechanical performance [163]. Furthermore, an antagomiR-188-conjugated aptamer targeting bone marrow mesenchymal stem cells (BMSCs) has been demonstrated to enhance bone formation and reduce fat accumulation in aged mice [164]. In other approaches, sclerostin, a glycoprotein secreted by osteocytes, plays a pivotal role in modulating bone formation by suppressing the osteogenic differentiation of mesenchymal stem cells or osteoprogenitor cells and the proliferation of osteoblasts [198]. Direct inhibition of sclerostin by using aptamers suggests that bone-targeted drug therapy could be an effective approach to treating osteoporosis. For example, Yuting Niu et al. developed a bone-targeting nanomedicine (DNA-MSN, DNAM) containing an anti-sclerostin aptamer (Aptscl56) immobilized on a PEGylated dendritic mesoporous silica nanoparticle (MSN) core, which exhibited high *in vivo* compatibility and a prolonged lifespan (Figure 6) [165]. Aptscl56 binds hydroxyapatite-bound calcium in bone, inhibiting sclerostin activity and restoring serum sclerostin levels and bone turnover to normal ranges. Overall, aptamer-based therapies for osteoporosis show great potential, although further studies are required to expand and validate strategies.

#### 4.2.6. Treatment of Cancer

Elevated BCL-2 levels due to aging increase cellular resistance to apoptosis, while age-related decline in DNA repair capacity contributes to long-term cancer risk [199]. In addition, SASP, an aging-related biomarker, significantly alters the tumor microenvironment and promotes tumorigenesis [200,201]. Notably, the resistance of anti-cancer drugs caused by aging presents a major hurdle in cancer treatment [199,202]. However, high drug doses to override drug resistance often lead to serious toxicity in normal tissues. In these situations, aptamers can be a powerful alternative drug with no side effects due to their high biocompatibility. Therefore, various aptamer-based cancer therapies have been developed through conjugation with functional materials, including drug payloads and nanomaterials [203].

**Figure 7:**
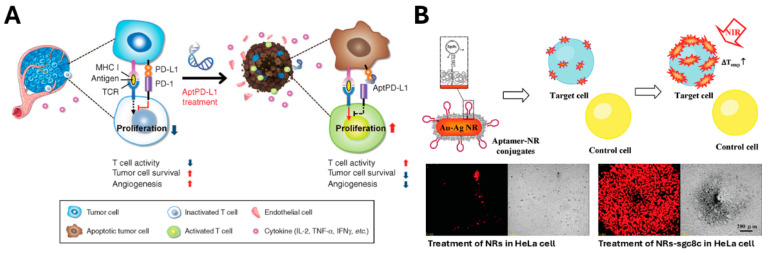
Aptamer-based therapeutic strategies for cancer. (**A**) Schematic illustration of an anti-cancer approach using the aptPD-L1 aptamer. This aptamer blocks PD-1/PD-L1 interactions and induces cytokine production (IL-2, TNF-α, IFN-γ, CXCL9, CXCL10), suppressing tumor angiogenesis and enhancing T cell function. Reprinted from [166]. (**B**) Schematic illustration of Aptamer-based photothermal cancer therapy using gold nanorods. The sgc8c aptamer targets HeLa cells, which are irradiated with 808 nm near-infrared light for 10 min. Cell death is confirmed via propidium iodide staining, demonstrating high selectivity and efficacy. Adapted with permission from [174]. Copyright (2008) American Chemical Society.

One approach involves enhancing T cell activation by inhibiting the immune evasion mechanisms of cancer cells, thereby allowing T cells to eliminate them. The binding of PD-1 and PD-L1 is a critical pathway in the immune circuitry of cancer cells, and several human cancer immunotherapies have been developed to block PD-1 and PD-L1 interaction [204]. Wei-Yun Lai et al. developed a PD-L1-targeting aptamer (aptPD-L1) that restored T cell function and inhibited tumor growth (Figure 7A) [166]. Another strategy leverages the elevated SASP environment in tumors. For instance, high IL-6 levels in cancer cells allow IL-6 receptor (IL-6R)-targeting aptamers to serve as delivery vehicles for chemotherapeutics [205,206,207]. A 5-fluoro-2′-deoxyuridine (5-FUdR)-conjugated IL-6R aptamer induced cancer cell-specific cytotoxicity by inhibiting DNA synthesis and inducing apoptosis [167]. Furthermore, nucleolin, overexpressed in many cancers, is another promising target for anticancer aptamer development [208]. Christopher R. Ireson et al. discovered AS1411, a nucleolin-targeting aptamer that specifically binds to the surface of cancer cells and then invades the inside of cancer cells [209,210]. AS1411 itself induces cancer cell death by inhibiting DNA replication and suppressing BCL-2 mRNA stabilization, thereby halting cell proliferation [168]. Exploiting this targeting capability, Huachao Chen et al. created an ATP-responsive delivery system using aptamers to release doxorubicin (DOX) selectively in the mitochondria of cancer [170], whereas Hang Xing et al. developed DOX-loaded liposomes conjugated with AS1411 to enhance tumor targeting [169]. Additionally, aptamer-based cancer therapies have incorporated diverse materials and mechanisms. These include silica nanoparticle-based drug delivery systems [211], platinum-encapsulated nanoparticles for direct tumor cell elimination [171], radiation-triggered photodynamic therapy via the photosensitizer chlorin e6 (Ce6) conjugation [172,173], and gold nanorod-coupled chemical and photothermal therapy using aptamer targeting [174,212] (Figure 7B). These strategies enable drug localization to tumor tissue while efficiently suppressing cancer cell proliferation.

## 5. Concluding Remarks and Outlook

Aging is a universal process influenced by genetic, environmental, and physiological factors, which contribute to the onset of age-related diseases and present societal challenges in an increasingly super-aged population. Numerous diagnostic and therapeutic strategies—based on both physiological and biochemical biomarkers—have been developed to monitor and mitigate the effects of aging. Recent advances in biosensing, stem cell therapy, and targeted treatments have further expanded these approaches. Notably, aptamers, due to their high stability, biocompatibility, compact size, ease of modification, conformational adaptability, and low immunogenicity, exhibit great potential in diagnostics and therapeutics. As a result, aptamers are emerging as major tools in the future of biomedical applications. This comprehensive review has explored the evolving landscape of aptamers and their role in addressing aging and age-related diseases.

Our review has shown that aptamers can serve as molecular recognition elements in biosensors, enabling highly specific and sensitive detection of biomarkers through optical and electrochemical platforms. In addition, beyond molecular targeting, aptamers have demonstrated their potential as therapeutic agents by modulating biomarkers of aging and age-related diseases. Importantly, aptamers can also facilitate targeted delivery to specific cells or organs and can even cross the blood–brain barrier to deliver therapeutic agents directly to the central nervous system. Therefore, aptamers, with their dual utility in diagnostics and therapeutics, have the potential to redefine current approaches to managing aging and age-related diseases.

Despite the versatility of aptamers, some challenges, such as the labor-intensive aptamer discovery process and off-target effects, have impeded the rapid response to new diseases and caused low diagnostic accuracy and treatment efficiency. However, these challenges can be easily solved by applying various advanced SELEX methods appropriate to the situation. In practice, methods such as magnetic bead-based SELEX, GO-SELEX, and particle display can minimize the time and cost for aptamer discovery by reducing the high-intensity iterations of conventional SELEX from 15 cycles to as low as 3 cycles. In addition, negative selection can be added to the SELEX process to discriminate between targets with similar structures, improving the specificity and sensitivity of the aptamers.

Furthermore, inherent limitations of oligonucleotides, such as susceptibility to nuclease degradation and rapid renal clearance, are regulatory hurdles that make it difficult to use the aptamer in clinical applications. To overcome these issues, many researchers are using chemical modification and conjugation strategies to enhance resistance to exonucleases and the half-life of *in vivo* circulation. For example, pegaptanib, the first aptamer-based drug registered with the FDA, was chemically modified by replacing the 2′-OH-group on purine nucleotides with 2′-O-methyl-groups, significantly prolonging its *in vivo* half-life [184]. Other xeno nucleic acid-based aptamers, such as peptide nucleic acids and locked nucleic acids, can also be engineered to maintain a long bioactive half-life while providing improved affinity [213,214,215]. Additionally, incorporation with metals such as gold and silver nanoparticles, carriers such as silica and exosomes, and DNA structures such as DNA tetrahedrons, circular DNA, and dimeric forms provide aptamers highly resistant to degradation through steric hindrance along with multi-functional features [216]. However, these chemical modification and conjugation strategies must be applied with caution. In particular, modified aptamers may carry unforeseen risks, including cytotoxicity due to accumulation, off-target activation, or induction of immune responses [217]. Therefore, appropriate dosage and rigorous evaluation are essential when introducing chemical modifications for therapeutic use.

In aptasensors, *in vitro* diagnostic methods employing patient samples are prevalent for disease detection rather than *in vivo* diagnostics. For example, an aptamer-functionalized nanochannel has been developed that can detect SARS-CoV-2 in COVID-19 patient samples in a single step [218]. This method utilizes aptamers that target the SARS-CoV-2 spike protein, enabling rapid and sensitive virus detection. Moreover, non-invasive diagnostic technologies have acquired considerable attention in the field of age-related diseases, particularly diabetes. For example, wearable electrochemical aptamer sensors can monitor insulin concentrations in real time, enabling more rapid and accurate diabetes management [219].

Even with significant advances, aptamer-based biosensors still face challenges, including inconsistent biofluid collection, electrode surface contamination, and physiological changes, in clinical trials. These issues can reduce sensing capacity, ensuring reliability, accuracy, and adaptability. In particular, the aging process involves a highly complex molecular network, and understanding the interplay among individual components remains a major challenge [32]. To address these challenges, efforts to define the aging phenotype continue through multiomics approaches aimed at identifying the hallmarks and biomarkers of aging and age-related diseases [220]. Moreover, advancing anti-aging research will require elucidating these mechanisms, and the application of cutting-edge technologies such as SOMAscan proteomics [221] and artificial intelligence (AI) [222] will be instrumental in addressing these complexities and improving predictive accuracy.

Finally, future applications of aptamers should consider the balance of the biosystem in complex biological environments, not just primary or secondary therapeutic approaches through simple target binding or delivery systems. In modern times, it is widely recognized that the body has its own circadian rhythms of body temperature, blood pressure, activity levels, and inflammatory responses. The proper regulation of these rhythms in accordance with the individual biological clock is critical for human health. In addition, aging and age-related diseases are closely related to the biological clock [223,224]. The circadian rhythm exerts both positive and negative effects on life expectancy, highlighting its importance in overall health and longevity. Proper circadian alignment contributes to physiological homeostasis and may delay the onset of age-associated conditions. In particular, rhythmic gene expression and the oscillatory activity of metabolic pathways are essential for maintaining the appropriate function of clock genes [225,226]. Modulation of circadian rhythm through lifestyle and behavioral interventions (intermittent fasting, maintaining consistent sleep–wake schedules, and consuming natural compounds that influence clock gene activity) offers a promising strategy for counteracting aging and associated disorders [28,227,228]. From this perspective, aptamer-based technologies could be a promising strategy for optimizing circadian rhythms in sleep regulation, food intake, exercise, and so on. For instance, melanopsin aptamers can reset the circadian phase of the biological clock, allowing for the maintenance of stable sleep activity [229]. Consequently, these approaches may indicate effective and safer solutions against age-related problems, focusing on the balance of biological systems rather than directly addressing disease-causing factors.

## Figures and Tables

**Table 1:** Comparison of aptamers and other molecules.

	Aptamer	Antibody	Small Molecule	Peptide
Size	~30 kDa	150~180 kDa	less than 1 kDa	~5 kDa
Affinity	nM-pM	nM-pM	μM-nM	μM-nM
Isolation process	*In vitro* process using SELEX (2–8 weeks)	*In vivo* biological production (weeks to months)	High-performance liquid chromatography (HPLC), Solid-phase extraction (SPE), Affinity purification (hours to days)	Ion-exchange chromatography, HPLC, lyophilization (hours to days)
Production cost	Low	High	Low	Low
Stability (pH, temperature)	Stable	Unstable	Stable	Unstable
Specificity	High	High	Low	Moderate
Chemical modification	Easy	Limited	Easy	Easy
Target potential	Proteins, peptides, cells, tissues, virus, bacteria, ions, small molecules	Proteins, peptides, cells, tissues, virus, bacteria, ions, small molecules	Proteins, enzymes, receptors, ion channels, DNA, RNA	Mainly proteins, peptides
*In vivo* half-life	Very short (minutes)	Long (days to weeks)	Variable (hours to days)	Short (minutes to hours)
Immunogenicity	Low	High	Low	Moderate
Cytotoxicity	Low	Variable	Variable	Variable
*In vivo* dosage	Low (μg-mg/mL)	High (mg/mL)	High (μg-mg/mL)	High (μg-mg/mL)

**Table 3:** The characteristics of aptamer-based biosensors.

Transducer		Mechanism	Sensitivity	Applicability
Optical	Fluorescence	Recognition of fluorescence signal change through aptamer conformational change	fM-pM	-
	Luminescence	Recognition of emission light from chemical reactions	fM-pM	*in vitro*
	Colorimetric	Recognition of color alterations	pM-nM	-
	Surface plasmon resonance (SPR)	Recognition of alterations in the refractive index	fM-pM	*in vitro*
	Biolayer interferometry (BLI)	Recognition of alterations in the interference pattern of light	pM-nM	-
	BLI-enzyme-linked aptamer sorbent assay (ELASA)	Highly sensitive detection, real-time monitoring by combining BLI and ELISA	fM	*in vitro*
	Enzyme-Linked Oligonucleotide Assay (ELONA)	Recognition of enzyme-substrate reactions	pM-nM	-
Electrochemical	Voltametric sensor	Recognition of current response of reaction between aptamers and molecules at electrode surface	fM-pM	*in vitro*
	Amperometric sensor	Recognition of current alterations by electrochemical reduction or oxidation of molecules at an electrode	pM-nM	-
	Impedance, Potentiometric sensor	Recognition of alterations in electrical potential or in impedance of the sensor surface	fM	-

**Table 5:** The list of aptamer-based therapeutics.

	Aptamer	Target	Therapeutic Principles	Ref.
Aging	Aptamin C	Vitamin C	Reduction of ROS levels by improving vitamin C half-life	[143]
	SIRT1 aptamer	Sirtuin1	Reduction of ROS levels by Sirtuin 1 activation	[144]
	yly12	Cell adhesion molecule L1	Induction of senescent cell apoptosis via aptamer-prodrug conjugation and SA-β-gal selective activity	[145,146]
Neurodegenerative diseases	Aβ7-92-1H1 (Aβ-Apt)	Aβ42 monomer	Inhibition of Aβ42 aggregation	[147]
	Tau-1 aptamer	Tau protein	Inhibition of tau protein aggregation	[148]
	F5R1, F5R2	α-syn	Inhibition of α-syn aggregation	[149]
			Inhibition of α-syn aggregation of *in vivo* neuronal cells via exosome-based F5R2 delivery	[150]
	Circular Tau–TfR bispecific aptamer	Tau protein +TfR	Blood-brain barrier permeation and tau aggregation inhibition via tau-transferrin receptor bifunctional aptamer	[151]
AMD	EYE001	VEGF	Blocking the binding of VEGF to the human VEGF receptor	[141,152]
	Pegaptanib			
	E10030	Platelet-derived growth factor (PDGF)	Inhibition of VEGF co-activation via PDGF receptor binding blockade	[153,154]
	RBM-007	Fibroblast growth factor 2 (FGF2)	Inhibition of FGF2 function	[155]
CVD	avβ3 aptamer	Integrin avβ3	Prevention of vascular smooth muscle cell (VSMC) proliferation and migration via suppression of Ras-phosphatidylinositol-4,5-bisphosphate 3-kinase/mitogen-activated protein kinase (PI3K/MAPK) signaling activity	[156]
	Apt14	VSMC	Inhibition of VSMC migration through activation of platelet-derived growth factor receptor (PDGFR)-β and inhibition of phosphatidylinositol 3-kinase/protein kinase-B (PI3K/Akt) signaling	[157]
	ARC1779	vWF	Inhibition of platelet aggregation and promotion of revascularization in platelet-rich thrombotic occlusions	[158]
	ARC1172			[159]
	DTRI-031			[160]
	BT200			[161]
	BC007	Autoantibodies against G-protein coupled receptors (GPCR-AABs)	Treatment of autoimmune-induced cardiomyopathy through neutralization of autoantibodies against G-protein coupled receptors (GPCR-AABs)	[162]
Osteoporosis	CH6	Rat and human osteoblasts	Promotion of bone formation and enhancing mechanical performance by delivery of pleckstrin homology domain-containing family O member 1 (Plekho1) siRNA-encapsulated nanoparticles	[163]
	BMSC-targeting aptamer	Bone marrow mesenchymal stem cells (BMSCs)	Increasing bone formation and decreasing fat accumulation through antagomiR-188 delivery to BMSCs	[164]
	Aptscl56	Sclerostin	Direct inhibition of sclerostin activity around hydroxyapatite via binding to bone calcium	[165]
Cancer	AptPD-L1	PD-L1	Restoration of T-cell function and inhibition of tumor growth by blockade of PD-1 and PD-L1 interactions	[166]
	AIR-3A	human IL-6 receptor (hIL-6R)	Inhibiting DNA biosynthesis and killing cancer cells via 5-fluoro-2′-deoxyuridine (5-FUdR) delivery	[167]
	AS1411	Nucleolin	Induction of cancer cell death by suppressing DNA replication and inhibition of cancer cell proliferation by disrupting stabilization of BCL-2 mRNA	[168]
			Elimination of cancer cells via delivery of DOX-loaded liposomes	[169]
	ATP aptamer +Cyt c aptamer +AS1411	ATP/cytochrome c/nucleolin	Elimination of cancer cells by releasing doxorubicin (DOX) under high ATP conditions in cancer cell mitochondria	[170]
	A10 RNA aptamer	Prostate-specific membrane antigen (PSMA)	Cancer treatment through delivery of a cytotoxic platinum-encapsulated nanoparticle	[171]
	TD05	Ramos cells	Radiation-induced photodynamic cancer therapy via delivery of the photosensitizer chlorine e6 (Ce6)	[172]
	Angiogenin aptamer	Human angiogenin		[173]
	sgc8	PTK7	Photothermal cancer therapy using gold nanorods	[174]

**Table 4:** The list of aptamer-based diagnoses and biosensors.

Transducer		Aptamer Name	Target	Limit of Detection (LOD)	Ref.
Optical	Fluorescence	IT3	p-tau231	3.64 ng/mL	[116]
	Luminescence	VEGF Apt1, VEGF Apt2	Vascular endothelial growth factor (VEGF)	1 ng/mL	[117]
	Colorimetric	pA-pT-apt	Amyloid-β1-40 oligomer (Aβ40-O)	3.03 nM	[118]
		Aβo, H1 aptamer	Amyloid-β oligomer (Aβo)	0.23 pM	[119]
	Surface plasmon resonance (SPR)	CRP-40-17, CRP-80-17	Human C-reactive protein (CRP)	0.35 nmol/L	[120]
		3′-thiol-modified 6th-62-40	CRP	10 pM	[121]
		HER2 aptamer	Human epidermal growth factor receptor 2 (HER2)-positive exosomes	10^4^–10^7^ particles/mL	[122]
	Biolayer interferometry (BLI)	Biotinylated DNA aptamer	Tau441 protein	6.7 nM	[123]
	BLI-ELASA	APT2TM	Growth differentiation factor-15 (GDF15)	5–6 pg/mL	[124]
	ELONA	Apt.1, Apt.2	Recombinant human cardiac troponin T isoform 6 (cTnT3)	3.42 nM, 3.13 nM	[125]
Electro-chemical	Square wave voltammetry (SWV)	cTnI aptamer	Cardiac troponin I (cTnI)	40 pg/mL	[125]
		NAD3-1a	NAD(H)	0.601 pM	[126]
		C10K2	Recombinant human osteopontin (rhOPN)	1.3 ± 0.1 nM	[127]
	Cyclic voltammetry (CV)	Amino-Apt13, Template-Apt12	VEGF	4.6 pmol/L	[128]
		Aβ-40-specific aptamer	Aß-40 peptides	20 fM	[129]
	Differential pulse voltammetry (DPV)	Thiol-functionalized aptamer Tro4	cTnI	2.03 fg/mL	[130]
		PD-L1 Apt., CD63 Apt.	Programmed cell death ligand 1 protein-positive (PD-L1+) exosomes	36 particles/mL	[131]
	Pulsed amperometric detection (PAD)	combSl2B, stalkGTG, r_stalkGTG	VEGF	0.21 nM	[132]
	Field-effect transistor (FET)	Anti-VEGF RNA aptamer (CPNT2-aptamer)	VEGF	400 fM	[133]

## Data Availability

Data are contained within the article.

## References

[1] United Nations World Population Prospects 2024: Summary of Results. https://population.un.org/wpp/assets/Files/WPP2024_Summary-of-Results.pdf.

[2] de Magalhaes J.P. (2024). Distinguishing between driver and passenger mechanisms of aging. Nat. Genet..

[3] Lopez-Otin C., Blasco M.A., Partridge L., Serrano M., Kroemer G. (2023). Hallmarks of aging: An expanding universe. Cell.

[4] Chen C., Ding S., Wang J. (2023). Digital health for aging populations. Nat. Med..

[5] Khan Y., Ostfeld A.E., Lochner C.M., Pierre A., Arias A.C. (2016). Monitoring of Vital Signs with Flexible and Wearable Medical Devices. Adv. Mater..

[6] Swaroop K.N., Chandu K., Gorrepotu R., Deb S. (2019). A health monitoring system for vital signs using IoT. Internet Things.

[7] Mensa E., Latini S., Ramini D., Storci G., Bonafe M., Olivieri F. (2019). The telomere world and aging: Analytical challenges and future perspectives. Ageing Res. Rev..

[8] Kim W., Shay J.W. (2018). Long-range telomere regulation of gene expression: Telomere looping and telomere position effect over long distances (TPE-OLD). Differentiation.

[9] Chang S., Multani A.S., Cabrera N.G., Naylor M.L., Laud P., Lombard D., Pathak S., Guarente L., DePinho R.A. (2004). Essential role of limiting telomeres in the pathogenesis of Werner syndrome. Nat. Genet..

[10] de Jesus B.B., Vera E., Schneeberger K., Tejera A.M., Ayuso E., Bosch F., Blasco M.A. (2012). Telomerase gene therapy in adult and old mice delays aging and increases longevity without increasing cancer. EMBO Mol. Med..

[11] Lee B.Y., Han J.A., Im J.S., Morrone A., Johung K., Goodwin E.C., Kleijer W.J., DiMaio D., Hwang E.S. (2006). Senescence-associated β-galactosidase is lysosomal β-galactosidase. Aging Cell.

[12] Safir Filho M., Dao P., Gesson M., Martin A.R., Benhida R. (2018). Development of highly sensitive fluorescent probes for the detection of beta-galactosidase activity—Application to the real-time monitoring of senescence in live cells. Analyst.

[13] Zhang S., Wang X., Wang X., Wang T., Liao W., Yuan Y., Chen G., Jia X. (2022). A novel AIE fluorescent probe for beta-galactosidase detection and imaging in living cells. Anal. Chim. Acta.

[14] Liu J., Ma X.W., Cui C., Chen Z.X., Wang Y., Deenik P.R., Cui L.N. (2021). Noninvasive NIR Imaging of Senescence *viaIn Situ* Labeling. J. Med. Chem..

[15] Cai Y., Zhou H., Zhu Y., Sun Q., Ji Y., Xue A., Wang Y., Chen W., Yu X., Wang L. (2020). Elimination of senescent cells by beta-galactosidase-targeted prodrug attenuates inflammation and restores physical function in aged mice. Cell Res..

[16] Munoz-Espin D., Rovira M., Galiana I., Gimenez C., Lozano-Torres B., Paez-Ribes M., Llanos S., Chaib S., Munoz-Martin M., Ucero A.C. (2018). A versatile drug delivery system targeting senescent cells. EMBO Mol. Med..

[17] Yang L., Liu G., Chen Q., Wan Y., Liu Z., Zhang J., Huang C., Xu Z., Li S., Lee C.S. (2022). An Activatable NIR Probe for the Detection and Elimination of Senescent Cells. Anal. Chem..

[18] de Magalhaes J.P., Stevens M., Thornton D. (2017). The Business of Anti-Aging Science. Trends Biotechnol..

[19] Ganceviciene R., Liakou A.I., Theodoridis A., Makrantonaki E., Zouboulis C.C. (2012). Skin anti-aging strategies. Dermatoendocrinology.

[20] Farooqui T., Farooqui A.A. (2009). Aging: An important factor for the pathogenesis of neurodegenerative diseases. Mech. Ageing Dev..

[21] Rodriguez M., Rodriguez-Sabate C., Morales I., Sanchez A., Sabate M. (2015). Parkinson’s disease as a result of aging. Aging Cell.

[22] Sengoku R. (2020). Aging and Alzheimer’s disease pathology. Neuropathology.

[23] Mitchell P., Liew G., Gopinath B., Wong T.Y. (2018). Age-related macular degeneration. Lancet.

[24] Fajemiroye J.O., da Cunha L.C., Saavedra-Rodriguez R., Rodrigues K.L., Naves L.M., Mourao A.A., da Silva E.F., Williams N.E.E., Martins J.L.R., Sousa R.B. (2018). Aging-Induced Biological Changes and Cardiovascular Diseases. BioMed Res. Int..

[25] Buford T.W. (2016). Hypertension and aging. Ageing Res. Rev..

[26] Yousufuddin M., Young N. (2019). Aging and ischemic stroke. Aging.

[27] Campisi J. (2013). Aging, cellular senescence, and cancer. Annu. Rev. Physiol..

[28] Ki M.R., Youn S., Kim D.H., Pack S.P. (2024). Natural Compounds for Preventing Age-Related Diseases and Cancers. Int. J. Mol. Sci..

[29] Huang Q., Tang J. (2010). Age-related hearing loss or presbycusis. Eur. Arch. Otorhinolaryngol..

[30] Pignolo R.J., Law S.F., Chandra A. (2021). Bone Aging, Cellular Senescence, and Osteoporosis. JBMR Plus.

[31] Han Y., Kim D.H., Pack S.P. (2024). Marine-Derived Bioactive Ingredients in Functional Foods for Aging: Nutritional and Therapeutic Perspectives. Mar. Drugs.

[32] Guo J., Huang X., Dou L., Yan M., Shen T., Tang W., Li J. (2022). Aging and aging-related diseases: From molecular mechanisms to interventions and treatments. Signal Transduct. Target. Ther..

[33] Glenner G.G., Wong C.W. (1984). Alzheimer’s disease: Initial report of the purification and characterization of a novel cerebrovascular amyloid protein. Biochem. Biophys. Res. Commun..

[34] Hardy J., Selkoe D.J. (2002). The amyloid hypothesis of Alzheimer’s disease: Progress and problems on the road to therapeutics. Science.

[35] Budde B., Schartner J., Tönges L., Kötting C., Nabers A., Gerwert K. (2019). Reversible Immuno-Infrared Sensor for the Detection of Alzheimer’s Disease Related Biomarkers. ACS Sens..

[36] Hsu C.H., Gupta A.K., Purwidyantri A., Prabowo B.A., Chen C.H., Chuang C.C., Tian Y.C., Lu Y.J., Lai C.S. (2022). Sensing Alzheimer’s Disease Utilizing Au Electrode by Controlling Nanorestructuring. Chemosensors.

[37] Adolfsson O., Pihlgren M., Toni N., Varisco Y., Buccarello A.L., Antoniello K., Lohmann S., Piorkowska K., Gafner V., Atwal J.K. (2012). An effector-reduced anti-beta-amyloid (Abeta) antibody with unique abeta binding properties promotes neuroprotection and glial engulfment of Abeta. J. Neurosci..

[38] Guarente L., Sinclair D.A., Kroemer G. (2024). Human trials exploring anti-aging medicines. Cell Metab..

[39] Dolton M.J., Chesterman A., Moein A., Sink K.M., Waitz A., Blondeau K., Kerchner G.A., Hu N., Brooks L., Roden A. (2021). Safety, Tolerability, and Pharmacokinetics of High-Volume Subcutaneous Crenezumab, With and Without Recombinant Human Hyaluronidase in Healthy Volunteers. Clin. Pharmacol. Ther..

[40] Yaku K., Okabe K., Nakagawa T. (2018). NAD metabolism: Implications in aging and longevity. Ageing Res. Rev..

[41] Mannick J.B., Lamming D.W. (2023). Targeting the biology of aging with mTOR inhibitors. Nat. Aging.

[42] Herdewijn P., Marlière P. (2009). Toward Safe Genetically Modified Organisms through the Chemical Diversification of Nucleic Acids. Chem. Biodivers..

[43] Ellington A.D., Szostak J.W. (1990). *In vitro* selection of RNA molecules that bind specific ligands. Nature.

[44] Park K.S., Cha H., Niu J., Soh H.T., Lee J.H., Pack S.P. (2024). DNA-controlled protein fluorescence: Design of aptamer-split peptide hetero-modulator for GFP to respond to intracellular ATP levels. Nucleic Acids Res..

[45] Park K.S., Park T.I., Lee J.E., Hwang S.Y., Choi A., Pack S.P. (2024). Aptamers and Nanobodies as New Bioprobes for SARS-CoV-2 Diagnostic and Therapeutic System Applications. Biosensors.

[46] Fu J., Yao F., An Y., Li X., Wang W., Yang X.-D. (2023). Novel bispecific aptamer targeting PD-1 and nucleolin for cancer immunotherapy. Cancer Nanotechnol..

[47] Rubahamya B., Dong S., Thurber G.M. (2024). Clinical translation of antibody drug conjugate dosing in solid tumors from preclinical mouse data. Sci. Adv..

[48] Odeh F., Nsairat H., Alshaer W., Ismail M.A., Esawi E., Qaqish B., Bawab A.A., Ismail S.I. (2019). Aptamers Chemistry: Chemical Modifications and Conjugation Strategies. Molecules.

[49] Jang E.K., Son R.G., Pack S.P. (2019). Novel enzymatic single-nucleotide modification of DNA oligomer: Prevention of incessant incorporation of nucleotidyl transferase by ribonucleotide-borate complex. Nucleic Acids Res..

[50] Park K.S., Choi A., Park T.I., Pack S.P. (2024). Fluorometric and Colorimetric Method for SARS-CoV-2 Detection Using Designed Aptamer Display Particles. Biosensors.

[51] Schmitt C.A., Wang B.S., Demaria M. (2022). Senescence and cancer—Role and therapeutic opportunities. Nat. Rev. Clin. Oncol..

[52] Tuerk C., Gold L. (1990). Systematic evolution of ligands by exponential enrichment: RNA ligands to bacteriophage T4 DNA polymerase. Science.

[53] Mendonsa S.D., Bowser M.T. (2004). *In vitro* evolution of functional DNA using capillary electrophoresis. J. Am. Chem. Soc..

[54] Bruno J.G. (1997). *In vitro* selection of DNA to chloroaromatics using magnetic microbead-based affinity separation and fluorescence detection. Biochem. Biophys. Res. Commun..

[55] Mayer G., Ahmed M.S., Dolf A., Endl E., Knolle P.A., Famulok M. (2010). Fluorescence-activated cell sorting for aptamer SELEX with cell mixtures. Nat. Protoc..

[56] Park J.W., Tatavarty R., Kim D.W., Jung H.T., Gu M.B. (2012). Immobilization-free screening of aptamers assisted by graphene oxide. Chem. Commun..

[57] Sefah K., Shangguan D., Xiong X., O’Donoghue M.B., Tan W. (2010). Development of DNA aptamers using Cell-SELEX. Nat. Protoc..

[58] Vijg J., Dong X. (2020). Pathogenic Mechanisms of Somatic Mutation and Genome Mosaicism in Aging. Cell.

[59] Blackburn E.H., Epel E.S., Lin J. (2015). Human telomere biology: A contributory and interactive factor in aging, disease risks, and protection. Science.

[60] Tedone E., Huang E., O’Hara R., Batten K., Ludlow A.T., Lai T.P., Arosio B., Mari D., Wright W.E., Shay J.W. (2019). Telomere length and telomerase activity in T cells are biomarkers of high-performing centenarians. Aging Cell.

[61] Oh E.S., Petronis A. (2021). Origins of human disease: The chrono-epigenetic perspective. Nat. Rev. Genet..

[62] Seale K., Horvath S., Teschendorff A., Eynon N., Voisin S. (2022). Making sense of the ageing methylome. Nat. Rev. Genet..

[63] Hipp M.S., Kasturi P., Hartl F.U. (2019). The proteostasis network and its decline in ageing. Nat. Rev. Mol. Cell Biol..

[64] Lipinski M.M., Zheng B., Lu T., Yan Z., Py B.F., Ng A., Xavier R.J., Li C., Yankner B.A., Scherzer C.R. (2010). Genome-wide analysis reveals mechanisms modulating autophagy in normal brain aging and in Alzheimer’s disease. Proc. Natl. Acad. Sci. USA.

[65] Kenyon C.J. (2010). The genetics of ageing. Nature.

[66] Amorim J.A., Coppotelli G., Rolo A.P., Palmeira C.M., Ross J.M., Sinclair D.A. (2022). Mitochondrial and metabolic dysfunction in ageing and age-related diseases. Nat. Rev. Endocrinol..

[67] Gorgoulis V., Adams P.D., Alimonti A., Bennett D.C., Bischof O., Bishop C., Campisi J., Collado M., Evangelou K., Ferbeyre G. (2019). Cellular Senescence: Defining a Path Forward. Cell.

[68] Ruzankina Y., Brown E.J. (2007). Relationships between stem cell exhaustion, tumour suppression and ageing. Br. J. Cancer.

[69] Rezazadeh S., Ellison-Hughes G.M. (2024). Editorial: Stem cell exhaustion in aging. Front. Aging.

[70] Fafian-Labora J.A., O’Loghlen A. (2020). Classical and Nonclassical Intercellular Communication in Senescence and Ageing. Trends Cell Biol..

[71] Baechle J.J., Chen N., Makhijani P., Winer S., Furman D., Winer D.A. (2023). Chronic inflammation and the hallmarks of aging. Mol. Metab..

[72] Haran J.P., McCormick B.A. (2021). Aging, Frailty, and the Microbiome-How Dysbiosis Influences Human Aging and Disease. Gastroenterology.

[73] Hou Y.J., Dan X.L., Babbar M., Wei Y., Hasselbalch S.G., Croteau D.L., Bohr V.A. (2019). Ageing as a risk factor for neurodegenerative disease. Nat. Rev. Neurol..

[74] Beharry C., Cohen L.S., Di J., Ibrahim K., Briffa-Mirabella S., Alonso A.D. (2014). Tau-induced neurodegeneration: Mechanisms and targets. Neurosci. Bull..

[75] Ingelsson M. (2016). Alpha-Synuclein Oligomers-Neurotoxic Molecules in Parkinson’s Disease and Other Lewy Body Disorders. Front. Neurosci..

[76] Clinton L.K., Blurton-Jones M., Myczek K., Trojanowski J.Q., LaFerla F.M. (2010). Synergistic Interactions between Aβ, Tau, and α-Synuclein: Acceleration of Neuropathology and Cognitive Decline. J. Neurosci..

[77] Goedert M., Spillantini M.G., Jakes R., Rutherford D., Crowther R.A. (1989). Multiple isoforms of human microtubule-associated protein tau: Sequences and localization in neurofibrillary tangles of Alzheimer’s disease. Neuron.

[78] Sengupta U., Kayed R. (2022). Amyloid beta, Tau, and alpha-Synuclein aggregates in the pathogenesis, prognosis, and therapeutics for neurodegenerative diseases. Prog. Neurobiol..

[79] Smith W., Assink J., Klein R., Mitchell P., Klaver C.C., Klein B.E., Hofman A., Jensen S., Wang J.J., de Jong P.T. (2001). Risk factors for age-related macular degeneration: Pooled findings from three continents. Ophthalmology.

[80] Jager R.D., Mieler W.F., Miller J.W. (2008). Age-related macular degeneration. N. Engl. J. Med..

[81] Nagineni C.N., Kommineni V.K., William A., Detrick B., Hooks J.J. (2012). Regulation of VEGF expression in human retinal cells by cytokines: Implications for the role of inflammation in age-related macular degeneration. J. Cell. Physiol..

[82] Droho S., Cuda C.M., Perlman H., Lavine J.A. (2021). Macrophage-derived interleukin-6 is necessary and sufficient for choroidal angiogenesis. Sci. Rep..

[83] Kushwah N., Bora K., Maurya M., Pavlovich M.C., Chen J. (2023). Oxidative Stress and Antioxidants in Age-Related Macular Degeneration. Antioxidants.

[84] Tang Y., Fung E., Xu A., Lan H.Y. (2017). C-reactive protein and ageing. Clin. Exp. Pharmacol. Physiol..

[85] Zhuang Q., Shen C., Chen Y., Zhao X., Wei P., Sun J., Ji Y., Chen X., Yang S. (2019). Association of high sensitive C-reactive protein with coronary heart disease: A Mendelian randomization study. BMC Med. Genet..

[86] Babuin L., Jaffe A.S. (2005). Troponin: The biomarker of choice for the detection of cardiac injury. CMAJ..

[87] Jenkins W.S., Vesey A.T., Stirrat C., Connell M., Lucatelli C., Neale A., Moles C., Vickers A., Fletcher A., Pawade T. (2017). Cardiac alpha(V)beta(3) integrin expression following acute myocardial infarction in humans. Heart.

[88] Spiel A.O., Gilbert J.C., Jilma B. (2008). von Willebrand factor in cardiovascular disease: Focus on acute coronary syndromes. Circulation.

[89] Kuo T.R., Chen C.H. (2017). Bone biomarker for the clinical assessment of osteoporosis: Recent developments and future perspectives. Biomark. Res..

[90] Rogers A., Eastell R. (2005). Circulating osteoprotegerin and receptor activator for nuclear factor kappaB ligand: Clinical utility in metabolic bone disease assessment. J. Clin. Endocrinol. Metab..

[91] Hofbauer L.C., Khosla S., Dunstan C.R., Lacey D.L., Boyle W.J., Riggs B.L. (2000). The roles of osteoprotegerin and osteoprotegerin ligand in the paracrine regulation of bone resorption. J. Bone Miner. Res..

[92] Faget D.V., Ren Q., Stewart S.A. (2019). Unmasking senescence: Context-dependent effects of SASP in cancer. Nat. Rev. Cancer.

[93] Kuilman T., Michaloglou C., Vredeveld L.C., Douma S., van Doorn R., Desmet C.J., Aarden L.A., Mooi W.J., Peeper D.S. (2008). Oncogene-induced senescence relayed by an interleukin-dependent inflammatory network. Cell.

[94] Takahashi A., Ohtani N., Yamakoshi K., Iida S., Tahara H., Nakayama K., Nakayama K.I., Ide T., Saya H., Hara E. (2006). Mitogenic signalling and the p16INK4a-Rb pathway cooperate to enforce irreversible cellular senescence. Nat. Cell Biol..

[95] Bent E.H., Gilbert L.A., Hemann M.T. (2016). A senescence secretory switch mediated by PI3K/AKT/mTOR activation controls chemoprotective endothelial secretory responses. Genes Dev..

[96] Cha J.H., Chan L.C., Li C.W., Hsu J.L., Hung M.C. (2019). Mechanisms Controlling PD-L1 Expression in Cancer. Mol. Cell.

[97] Garo L.P., Ajay A.K., Fujiwara M., Gabriely G., Raheja R., Kuhn C., Kenyon B., Skillin N., Kadowaki-Saga R., Saxena S. (2021). MicroRNA-146a limits tumorigenic inflammation in colorectal cancer. Nat. Commun..

[98] Polymeropoulos M.H., Lavedan C., Leroy E., Ide S.E., Dehejia A., Dutra A., Pike B., Root H., Rubenstein J., Boyer R. (1997). Mutation in the alpha-synuclein gene identified in families with Parkinson’s disease. Science.

[99] Ferrara N., Mass R.D., Campa C., Kim R. (2007). Targeting VEGF-A to treat cancer and age-related macular degeneration. Annu. Rev. Med..

[100] Ye F., Kaneko H., Hayashi Y., Takayama K., Hwang S.J., Nishizawa Y., Kimoto R., Nagasaka Y., Tsunekawa T., Matsuura T. (2016). Malondialdehyde induces autophagy dysfunction and VEGF secretion in the retinal pigment epithelium in age-related macular degeneration. Free Radic. Biol. Med..

[101] Xiao R., Lei C., Zhang Y., Zhang M. (2023). Interleukin-6 in retinal diseases: From pathogenesis to therapy. Exp. Eye Res..

[102] Zhang S., Zhang Q., Lu Y., Chen J., Liu J., Li Z., Xie Z. (2024). Roles of Integrin in Cardiovascular Diseases: From Basic Research to Clinical Implications. Int. J. Mol. Sci..

[103] Singh S., Kumar D., Lal A.K. (2015). Serum osteocalcin as a diagnostic biomarker for primary osteoporosis in women. J. Clin. Diagn. Res. JCDR.

[104] Krege J.H., Lane N.E., Harris J.M., Miller P.D. (2014). PINP as a biological response marker during teriparatide treatment for osteoporosis. Osteoporos. Int..

[105] Hassager C., Jensen L.T., Johansen J.S., Riis B.J., Melkko J., Podenphant J., Risteli L., Christiansen C., Risteli J. (1991). The Carboxy-Terminal Propeptide of Type-I Procollagen in Serum as a Marker of Bone-Formation—The Effect of Nandrolone Decanoate and Female Sex-Hormones. Metab.-Clin. Exp..

[106] Baim S., Miller P.D. (2009). Assessing the Clinical Utility of Serum CTX in Postmenopausal Osteoporosis and Its Use in Predicting Risk of Osteonecrosis of the Jaw. J. Bone Miner. Res..

[107] Yamagishi S. (2011). Role of advanced glycation end products (AGEs) in osteoporosis in diabetes. Curr. Drug Targets.

[108] Gaudio A., Pennisi P., Bratengeier C., Torrisi V., Lindner B., Mangiafico R.A., Pulvirenti I., Hawa G., Tringali G., Fiore C.E. (2010). Increased sclerostin serum levels associated with bone formation and resorption markers in patients with immobilization-induced bone loss. J. Clin. Endocrinol. Metab..

[109] Krishnamurthty J., Torrice C., Ramsey M.R., Kovalev G.I., Al-Regaiey K., Su L.S., Sharpless N.E. (2004). Ink4a/Arf expression is a biomarker of aging. J. Clin. Investig..

[110] Zou W., Wolchok J.D., Chen L. (2016). PD-L1 (B7-H1) and PD-1 pathway blockade for cancer therapy: Mechanisms, response biomarkers, and combinations. Sci. Transl. Med..

[111] Jiang G., Zhang M., Yue B., Yang M., Carter C., Al-Quran S.Z., Li B., Li Y. (2012). PTK7: A new biomarker for immunophenotypic characterization of maturing T cells and T cell acute lymphoblastic leukemia. Leuk. Res..

[112] Song S.P., Wang L.H., Li J., Zhao J.L., Fan C.H. (2008). Aptamer-based biosensors. TrAC-Trends Anal. Chem..

[113] Zhu G., Chen X. (2018). Aptamer-based targeted therapy. Adv. Drug. Deliv. Rev..

[114] Chen C., Zhou M., Ge Y.C., Wang X.B. (2020). SIRT1 and aging related signaling pathways. Mech. Ageing Dev..

[115] Yamagishi S.I., Matsui T. (2018). Therapeutic Potential of DNA-aptamers Raised Against AGE-RAGE Axis in Diabetes-related Complications. Curr. Pharm. Des..

[116] Phan L.M.T., Cho S. (2022). Fluorescent Aptasensor and Colorimetric Aptablot for p-tau231 Detection: Toward Early Diagnosis of Alzheimer’s Disease. Biomedicines.

[117] Shan S., He Z., Mao S., Jie M., Yi L., Lin J.M. (2017). Quantitative determination of VEGF165 in cell culture medium by aptamer sandwich based chemiluminescence assay. Talanta.

[118] Tu Y., Wu J., Chai K., Hu X., Hu Y., Shi S., Yao T. (2023). A turn-on unlabeled colorimetric biosensor based on aptamer-AuNPs conjugates for amyloid-beta oligomer detection. Talanta.

[119] Chen M., Man Y., Xu S., Wu H., Ling P., Gao F. (2023). A label-free dually-amplified aptamer sensor for the specific detection of amyloid-beta peptide oligomers in cerebrospinal fluids. Anal. Chim. Acta.

[120] Yang X.H., Wang Y.N., Wang K.M., Wang Q., Wang P., Lin M., Chen N., Tan Y.Y. (2014). DNA aptamer-based surface plasmon resonance sensing of human C-reactive protein. RSC Adv..

[121] Wu B., Jiang R., Wang Q., Huang J., Yang X., Wang K., Li W., Chen N., Li Q. (2016). Detection of C-reactive protein using nanoparticle-enhanced surface plasmon resonance using an aptamer-antibody sandwich assay. Chem. Commun..

[122] Chen W., Li Z., Cheng W., Wu T., Li J., Li X., Liu L., Bai H., Ding S., Li X. (2021). Surface plasmon resonance biosensor for exosome detection based on reformative tyramine signal amplification activated by molecular aptamer beacon. J. Nanobiotechnology.

[123] Ziu I., Laryea E.T., Alashkar F., Wu C.G., Martic S. (2020). A dip-and-read optical aptasensor for detection of tau protein. Anal. Bioanal. Chem..

[124] Gao S., Li Q., Zhang S., Sun X., Zhou H., Wang Z., Wu J. (2023). A novel biosensing platform for detection of glaucoma biomarker GDF15 via an integrated BLI-ELASA strategy. Biomaterials.

[125] Torrini F., Palladino P., Brittoli A., Baldoneschi V., Minunni M., Scarano S. (2019). Characterization of troponin T binding aptamers for an innovative enzyme-linked oligonucleotide assay (ELONA). Anal. Bioanal. Chem..

[126] Guo W., Wang H., Wang Z., Wu F., He Y., Liu Y., Deng Y., Bing T., Qiu L., Tan W. (2025). DNA aptamer-based sensitive electrochemical biosensor for NAD(H) detection. Biosens. Bioelectron..

[127] Meirinho S.G., Dias L.G., Peres A.M., Rodrigues L.R. (2017). Electrochemical aptasensor for human osteopontin detection using a DNA aptamer selected by SELEX. Anal. Chim. Acta.

[128] Fu X.M., Liu Z.J., Cai S.X., Zhao Y.P., Wu D.Z., Li C.Y., Chen J.H. (2016). Electrochemical aptasensor for the detection of vascular endothelial growth factor (VEGF) based on DNA-templated Ag/Pt bimetallic nanoclusters. Chin. Chem. Lett..

[129] Kutovyi Y., Hlukhova H., Boichuk N., Menger M., Offenhausser A., Vitusevich S. (2020). Amyloid-beta peptide detection via aptamer-functionalized nanowire sensors exploiting single-trap phenomena. Biosens. Bioelectron..

[130] Jiang L., Li D., Su M., Qiu Y., Chen F., Qin X., Wang L., Gui Y., Zhao J., Guo H. (2024). A Label-Free Electrochemical Aptamer Sensor for Sensitive Detection of Cardiac Troponin I Based on AuNPs/PB/PS/GCE. Nanomaterials.

[131] Chang L., Wu H., Chen R., Sun X., Yang Y., Huang C., Ding S., Liu C., Cheng W. (2023). Microporous PdCuB nanotag-based electrochemical aptasensor with Au@CuCl_2_ nanowires interface for ultrasensitive detection of PD-L1-positive exosomes in the serum of lung cancer patients. J. Nanobiotechnol..

[132] Liustrovaite V., Ratautaite V., Ramanaviciene A., Plikusiene I., Malinovskis U., Erts D., Sarvutiene J., Ramanavicius A. (2024). Electrochemical sensor for vascular endothelial growth factor based on self-assembling DNA aptamer structure. Sci. Total Environ..

[133] Kwon O.S., Park S.J., Jang J. (2010). A high-performance VEGF aptamer functionalized polypyrrole nanotube biosensor. Biomaterials.

[134] Peltomaa R., Glahn-Martinez B., Benito-Pena E., Moreno-Bondi M.C. (2018). Optical Biosensors for Label-Free Detection of Small Molecules. Sensors.

[135] Napit R., Jaysawal S.K., Chowdhury R., Catague J., Melke H., Pham C.V., Xu H., Jia L., Lin J., Hou Y.C. (2025). Aptasensors and Advancement in Molecular Recognition Technology. Adv. Mater. Technol..

[136] Downs A.M., Gerson J., Leung K.K., Honeywell K.M., Kippin T., Plaxco K.W. (2022). Improved calibration of electrochemical aptamer-based sensors. Sci. Rep..

[137] Mei C., Zhang Y., Pan L., Dong B., Chen X., Gao Q., Xu H., Xu W., Fang H., Liu S. (2022). A One-Step Electrochemical Aptasensor Based on Signal Amplification of Metallo Nanoenzyme Particles for Vascular Endothelial Growth Factor. Front. Bioeng. Biotechnol..

[138] Mikula E., Malecka-Baturo K. (2023). An Overview of the Latest Developments in the Electrochemical Aptasensing of Neurodegenerative Diseases. Coatings.

[139] Liu Y.X., Dykstra G. (2022). Recent progress on electrochemical (bio)sensors based on aptamer-molecularly imprinted polymer dual recognition. Sens. Actuator Rep..

[140] Lang M., Luo D., Yang G., Mei Q., Feng G., Yang Y., Liu Z., Chen Q., Wu L. (2020). An ultrasensitive electrochemical sensing platform for the detection of cTnI based on aptamer recognition and signal amplification assisted by TdT. RSC Adv..

[141] Gragoudas E.S., Adamis A.P., Cunningham E.T., Feinsod M., Guyer D.R., VEGF Inhibition Study in Ocular Neovascularization Clinical Trial Group (2004). Pegaptanib for neovascular age-related macular degeneration. N. Engl. J. Med..

[142] Kang C. (2023). Avacincaptad Pegol: First Approval. Drugs.

[143] Choi S., Han J., Kim J.H., Kim A.R., Kim S.H., Lee W., Yoon M.Y., Kim G., Kim Y.S. (2020). Advances in dermatology using DNA aptamer “Aptamin C” innovation: Oxidative stress prevention and effect maximization of vitamin C through antioxidation. J. Cosmet. Dermatol..

[144] Salman R.F., Al-Sudani B.T., Mshimesh B.A.R. (2023). Protective Action of SIRT1 Activator Aptamer in Human Skin Cell Line. J. Popul. Ther. Clin. Pharmacol..

[145] Xia Y., Li J., Wang L., Xie Y., Zhang L., Han X., Tan W., Liu Y. (2023). Engineering Hierarchical Recognition-Mediated Senolytics for Reliable Regulation of Cellular Senescence and Anti-Atherosclerosis Therapy. Angew. Chem. Int. Ed. Engl..

[146] Xie Y., Li J., Wu P., Wang L., Hong D., Wang J., Liu Y. (2023). Targeted regulation of senescence-associated secretory phenotype with an aptamer-conjugated activatable senomorphic. Aging Pathobiol. Ther..

[147] Zheng Y., Wang P., Li S.Y., Geng X.H., Zou L.Y., Jin M.M., Zou Q.Q., Wang Q., Yang X.H., Wang K.M. (2020). Development of DNA Aptamer as a β-Amyloid Aggregation Inhibitor. ACS Appl. Bio Mater..

[148] Kim J.H., Kim E., Choi W.H., Lee J., Lee J.H., Lee H., Kim D.E., Suh Y.H., Lee M.J. (2016). Inhibitory RNA Aptamers of Tau Oligomerization and Their Neuroprotective Roles against Proteotoxic Stress. Mol. Pharm..

[149] Zheng Y., Qu J., Xue F.Q., Zheng Y., Yang B., Chang Y.C., Yang H., Zhang J.L. (2018). Novel DNA Aptamers for Parkinson’s Disease Treatment Inhibit α-Synuclein Aggregation and Facilitate its Degradation. Mol. Ther.-Nucl. Acids.

[150] Ren X.X., Zhao Y., Xue F.Q., Zheng Y., Huang H.X., Wang W., Chang Y.C., Yang H., Zhang J.L. (2019). Exosomal DNA Aptamer Targeting α-Synuclein Aggregates Reduced Neuropathological Deficits in a Mouse Parkinson’s Disease Model. Mol. Ther.-Nucl. Acids.

[151] Li X.W., Yang Y., Zhao H.Z., Zhu T., Yang Z.H., Xu H.Y., Fu Y.Q., Lin F., Pan X.S., Li L. (2020). Enhanced *in Vivo* Blood-Brain Barrier Penetration by Circular Tau-Transferrin Receptor Bifunctional Aptamer for Tauopathy Therapy. J. Am. Chem. Soc..

[152] Martin D.F., Klein M., Haller J., Adamis A., Gragoudas E., Miller J., Blumenkrantz M., Goldberg M., Yannuzzi L., Henninger D. (2002). Preclinical and phase 1A clinical evaluation of an anti-VEGF pegylated aptamer (EYE001) for the treatment of exudative age-related macular degeneration. Retina.

[153] Akiyama H., Kachi S., Silva R.L.E., Umeda N., Hackett S.F., McCauley D., McCauley T., Zoltoski A., Epstein D.M., Campochiaro P.A. (2006). Intraocular injection of an aptamer that binds PDGF-B: A potential treatment for proliferative retinopathies. J. Cell. Physiol..

[154] Jaffe G.J., Eliott D., Wells J.A., Prenner J.L., Papp A., Patel S. (2016). A Phase 1 Study of Intravitreous E10030 in Combination with Ranibizumab in Neovascular Age-Related Macular Degeneration. Ophthalmology.

[155] Matsuda Y., Nonaka Y., Futakawa S., Imai H., Akita K., Nishihata T., Fujiwara M., Ali Y., Bhisitkul R.B., Nakamura Y. (2019). Anti-Angiogenic and Anti-Scarring Dual Action of an Anti-Fibroblast Growth Factor 2 Aptamer in Animal Models of Retinal Disease. Mol. Ther.-Nucl. Acids.

[156] Wu H.B., Wang Z.W., Shi F., Ren Z.L., Li L.C., Hu X.P., Hu R., Li B.W. (2020). Avbeta3 Single-Stranded DNA Aptamer Attenuates Vascular Smooth Muscle Cell Proliferation and Migration via Ras-PI3K/MAPK Pathway. Cardiovasc. Ther..

[157] Thiel W.H., Esposito C.L., Dickey D.D., Dassie J.P., Long M.E., Adam J., Streeter J., Schickling B., Takapoo M., Flenker K.S. (2016). Smooth Muscle Cell-targeted RNA Aptamer Inhibits Neointimal Formation. Mol. Ther..

[158] Gilbert J.C., DeFeo-Fraulini T., Hutabarat R.M., Horvath C.J., Merlino P.G., Marsh H.N., Healy J.M., BouFakhreddine S., Holohan T.V., Schaub R.G. (2007). First-in-human evaluation of anti-von Willebrand factor therapeutic aptamer ARC1779 in healthy volunteers. Circulation.

[159] Huang R.H., Fremont D.H., Diener J.L., Schaub R.G., Sadler J.E. (2009). A Structural Explanation for the Antithrombotic Activity of ARC1172, a DNA Aptamer that Binds von Willebrand Factor Domain A1. Structure.

[160] Nimjee S.M., Dornbos D., Pitoc G.A., Wheeler D.G., Layzer J.M., Venetos N., Huttinger A., Talentino S.E., Musgrave N.J., Moody H. (2019). Preclinical Development of a vWF Aptamer to Limit Thrombosis and Engender Arterial Recanalization of Occluded Vessels. Mol. Ther..

[161] Zhu S.H., Gilbert J.C., Hatala P., Harvey W., Liang Z.C., Gao S., Kang D.W., Jilma B. (2020). The development and characterization of a long acting anti-thrombotic von Willebrand factor (VWF) aptamer. J. Thromb. Haemost..

[162] Wallukat G., Müller J., Haberland A., Berg S., Schulz A., Freyse E.J., Vetter R., Salzsieder E., Kreutz R., Schimke I. (2016). Aptamer BC007 for neutralization of pathogenic autoantibodies directed against G-protein coupled receptors: A vision of future treatment of patients with cardiomyopathies and positivity for those autoantibodies. Atherosclerosis.

[163] Liang C., Guo B.S., Wu H., Shao N.S., Li D.F., Liu J., Dang L., Wang C., Li H., Li S.H. (2015). Aptamer-functionalized lipid nanoparticles targeting osteoblasts as a novel RNA interference-based bone anabolic strategy. Nat. Med..

[164] Li C.J., Cheng P., Liang M.K., Chen Y.S., Lu Q., Wang J.Y., Xia Z.Y., Zhou H.D., Cao X., Xie H. (2015). MicroRNA-188 regulates age-related switch between osteoblast and adipocyte differentiation. J. Clin. Investig..

[165] Niu Y.T., Yang Y., Yang Z., Wang X., Zhang P., Lv L.W., Liu Y., Liu Y.S., Zhou Y.S. (2022). Aptamer-immobilized bone-targeting nanoparticles in situ reduce sclerostin for osteoporosis treatment. Nano Today.

[166] Lai W.Y., Huang B.T., Wang J.W., Lin P.Y., Yang P.C. (2016). A Novel PD-L1-targeting Antagonistic DNA Aptamer With Antitumor Effects. Mol. Ther. Nucleic Acids.

[167] Kruspe S., Hahn U. (2014). An Aptamer Intrinsically Comprising 5-Fluoro-2′-deoxyuridine for Targeted Chemotherapy. Angew. Chem. Int. Ed..

[168] Soundararajan S., Chen W.W., Spicer E.K., Courtenay-Luck N., Fernandes D.J. (2008). The nucleolin targeting aptamer AS1411 destabilizes *Bcl.-2* messenger RNA in human breast cancer cells. Cancer Res..

[169] Xing H., Tang L., Yang X., Hwang K., Wang W., Yin Q., Wong N.Y., Dobrucki L.W., Yasui N., Katzenellenbogen J.A. (2013). Selective Delivery of an Anticancer Drug with Aptamer-Functionalized Liposomes to Breast Cancer Cells *in Vitro* and *in Vivo*. J. Mater. Chem. B.

[170] Chen H.C., Tian J.W., Liu D.Y., He W.J., Guo Z.J. (2017). Dual aptamer modified dendrigraft poly-L-lysine nanoparticles for overcoming multi-drug resistance through mitochondrial targeting. J. Mater. Chem. B.

[171] Dhar S., Gu F.X., Langer R., Farokhzad O.C., Lippard S.J. (2008). Targeted delivery of cisplatin to prostate cancer cells by aptamer functionalized Pt(IV) prodrug-PLGA-PEG nanoparticles. Proc. Natl. Acad. Sci. USA.

[172] Mallikaratchy P., Tang Z.W., Tan W.H. (2008). Cell specific aptamer-photosensitizer conjugates as a molecular tool in photodynamic therapy. Chemmedchem.

[173] Yang X., Huang J., Wang K., Li W., Cui L., Li X. (2011). Angiogenin-mediated photosensitizer-aptamer conjugate for photodynamic therapy. ChemMedChem.

[174] Huang Y.F., Sefah K., Bamrungsap S., Chang H.T., Tan W. (2008). Selective photothermal therapy for mixed cancer cells using aptamer-conjugated nanorods. Langmuir.

[175] Maldonado E., Morales-Pison S., Urbina F., Solari A. (2023). Aging Hallmarks and the Role of Oxidative Stress. Antioxidants.

[176] Lee J.M., Lee J.H., Kim S.H., Sim T.H., Kim Y.J. (2023). NXP032 ameliorates cognitive impairment by alleviating the neurovascular aging process in aged mouse brain. Sci. Rep..

[177] Li X., Li C., Zhang W., Wang Y., Qian P., Huang H. (2023). Inflammation and aging: Signaling pathways and intervention therapies. Signal Transduct. Target. Ther..

[178] Lai W.Y., Wang J.W., Huang B.T., Lin E.P.Y., Yang P.C. (2019). A Novel TNF-α-Targeting Aptamer for TNF-α-Mediated Acute Lung Injury and Acute Liver Failure. Theranostics.

[179] Zhang M., Wen Y.T., Huang Z.H., Qin X., Zhou M., Xiao D.X., Cui W.T., Liu Z.Q., Lin Y.F. (2023). Targeted therapy for autoimmune diseases based on multifunctional frame nucleic acid system: Blocking TNF-α-NF-ΚB signaling and mediating macrophage polarization. Chem. Eng. J..

[180] Cheng C.S., Chen Y.H., Lennox K.A., Behlke M.A., Davidson B.L. (2013). SELEX for Identification of Brain-penetrating Aptamers. Mol. Ther.-Nucl. Acids.

[181] Choi J.W., Seo M., Kim K., Kim A.R., Lee H., Kim H.S., Park C.G., Cho S.W., Kang J.H., Joo J. (2023). Aptamer Nanoconstructs Crossing Human Blood-Brain Barrier Discovered via Microphysiological System-Based SELEX Technology. ACS Nano.

[182] Alvarez-Erviti L., Seow Y.Q., Yin H.F., Betts C., Lakhal S., Wood M.J.A. (2011). Delivery of siRNA to the mouse brain by systemic injection of targeted exosomes. Nat. Biotechnol..

[183] Jellinek D., Green L.S., Bell C., Janjic N. (1994). Inhibition of Receptor-Binding by High-Affinity Rna Ligands to Vascular Endothelial Growth-Factor. Biochemistry.

[184] Ruckman J., Green L.S., Beeson J., Waugh S., Gillette W.L., Henninger D.D., Claesson-Welsh L., Janjic N. (1998). 2′-fluoropyrimidine RNA-based aptamers to the 165-amino acid form of vascular endothelial growth factor (VEGF165): Inhibition of receptor binding and VEGF-induced vascular permeability through interactions requiring the exon 7-encoded domain. J. Biol. Chem..

[185] Brown D.M., Kaiser P.K., Michels M., Soubrane G., Heier J.S., Kim R.Y., Sy J.P., Schneider S., Grp A.S. (2006). Ranibizumab versus verteporfin for neovascular age-related macular degeneration. N. Engl. J. Med..

[186] Heier J.S., Brown D.M., Chong V., Korobelnik J.F., Kaiser P.K., Nguyen Q.D., Kirchhof B., Ho A., Ogura Y., Yancopoulos G.D. (2012). Intravitreal Aflibercept (VEGF Trap-Eye) in Wet Age-related Macular Degeneration. Ophthalmology.

[187] Martin D.F., Maguire M.G., Fine S.L., Ying G.S., Jaffe G.J., Grunwald J.E., Toth C., Redford M., Ferris F.L., Deg C.A.-r.M. (2020). Ranibizumab and Bevacizumab for Treatment of Neovascular Age-related Macular Degeneration. Ophthalmology.

[188] Rofagha S., Bhisitkul R.B., Boyer D.S., Sadda S.R., Zhang K., Group S.-U.S. (2013). Seven-year outcomes in ranibizumab-treated patients in ANCHOR, MARINA, and HORIZON: A multicenter cohort study (SEVEN-UP). Ophthalmology.

[189] Bhisitkul R.B., Desai S.J., Boyer D.S., Sadda S.R., Zhang K. (2016). Fellow Eye Comparisons for 7-Year Outcomes in Ranibizumab-Treated AMD Subjects from ANCHOR, MARINA, and HORIZON (SEVEN-UP Study). Ophthalmology.

[190] Maguire M.G., Martin D.F., Ying G.S., Jaffe G.J., Daniel E., Grunwald J.E., Toth C.A., Ferris F., Fine S.L., Macular C.A.-r. (2016). Five-Year Outcomes with Anti-Vascular Endothelial Growth Factor Treatment of Neovascular Age-Related Macular Degeneration: The Comparison of Age-Related Macular Degeneration Treatments Trials. Ophthalmology.

[191] Mehta H., Tufail A., Daien V., Lee A.Y., Nguyen V., Ozturk M., Barthelmes D., Gillies M.C. (2018). Real-world outcomes in patients with neovascular age-related macular degeneration treated with intravitreal vascular endothelial growth factor inhibitors. Prog. Retin. Eye Res..

[192] Chen X.Y., Ma Y., Xie Y.Q., Pu J. (2022). Aptamer-based applications for cardiovascular disease. Front. Bioeng. Biotechnol..

[193] Thiel W.H., Bair T., Peek A.S., Liu X., Dassie J., Stockdale K.R., Behlke M.A., Miller F.J., Giangrande P.H. (2012). Rapid identification of cell-specific, internalizing RNA aptamers with bioinformatics analyses of a cell-based aptamer selection. PLoS ONE.

[194] Udofot O., Lin L.H., Thiel W.H., Erwin M., Turner E., Miller F.J., Giangrande P.H., Yazdani S.K. (2019). Delivery of Cell-Specific Aptamers to the Arterial Wall with an Occlusion Perfusion Catheter. Mol. Ther. Nucleic Acids.

[195] Kovacevic K.D., Greisenegger S., Langer A., Gelbenegger G., Buchtele N., Pabinger I., Petroczi K., Zhu S.H., Gilbert J.C., Jilma B. (2021). The aptamer BT200 blocks von Willebrand factor and platelet function in blood of stroke patients. Sci. Rep..

[196] Wallukat G., Schimke I. (2014). Agonistic autoantibodies directed against G-protein-coupled receptors and their relationship to cardiovascular diseases. Semin. Immunopathol..

[197] Kennel K.A., Drake M.T. (2009). Adverse Effects of Bisphosphonates: Implications for Osteoporosis Management. Mayo Clin. Proc..

[198] Ota K., Quint P., Ruan M., Pederson L., Westendorf J.J., Khosla S., Oursler M.J. (2013). Sclerostin is expressed in osteoclasts from aged mice and reduces osteoclast-mediated stimulation of mineralization. J. Cell. Biochem..

[199] Rysanek D., Vasicova P., Kolla J.N., Sedlak D., Andera L., Bartek J., Hodny Z. (2022). Synergism of BCL-2 family inhibitors facilitates selective elimination of senescent cells. Aging.

[200] Morales-Valencia J., Lau L., Marti-Nin T., Ozerdem U., David G. (2022). Therapy-induced senescence promotes breast cancer cells plasticity by inducing Lipocalin-2 expression. Oncogene.

[201] Jo H., Shim K., Jeoung D. (2023). The Potential of Senescence as a Target for Developing Anticancer Therapy. Int. J. Mol. Sci..

[202] Palazzo A., Hernandez-Vargas H., Goehrig D., Médard J.J., Vindrieux D., Flaman J.M., Bernard D. (2022). Transformed cells after senescence give rise to more severe tumor phenotypes than transformed non-senescent cells. Cancer Lett..

[203] Wu X., Chen J., Wu M., Zhao J.X. (2015). Aptamers: Active targeting ligands for cancer diagnosis and therapy. Theranostics.

[204] Intlekofer A.M., Thompson C.B. (2013). At the Bench: Preclinical rationale for CTLA-4 and PD-1 blockade as cancer immunotherapy. J. Leukoc. Biol..

[205] Meyer C., Eydeler K., Magbanua E., Zivkovic T., Piganeau N., Lorenzen I., Grötzinger J., Mayer G., Rose-John S., Hahn U. (2012). Interleukin-6 receptor specific RNA aptamers for cargo delivery into target cells. RNA Biol..

[206] Mittelberger F., Meyer C., Waetzig G.H., Zacharias M., Valentini E., Svergun D.I., Berg K., Lorenzen I., Grötzinger J., Rose-John S. (2015). RAID3-An interleukin-6 receptor-binding aptamer with post-selective modification-resistant affinity. RNA Biol..

[207] Hahn U. (2017). Charomers-Interleukin-6 Receptor Specific Aptamers for Cellular Internalization and Targeted Drug Delivery. Int. J. Mol. Sci..

[208] Thongchot S., Aksonnam K., Thuwajit P., Yenchitsomanus P.T., Thuwajit C. (2023). Nucleolin-based targeting strategies in cancer treatment: Focus on cancer immunotherapy (Review). Int. J. Mol. Med..

[209] Ireson C.R., Kelland L.R. (2006). Discovery and development of anticancer aptamers. Mol. Cancer Ther..

[210] Soundararajan S., Wang L., Sridharan V., Chen W.W., Courtenay-Luck N., Jones D., Spicer E.K., Fernandes D.J. (2009). Plasma Membrane Nucleolin Is a Receptor for the Anticancer Aptamer AS1411 in MV4-11 Leukemia Cells. Mol. Pharmacol..

[211] Aravind A., Veeranarayanan S., Poulose A.C., Nair R., Nagaoka Y., Yoshida Y., Maekawa T., Kumar D.S. (2012). Aptamer-functionalized silica nanoparticles for targeted cancer therapy. BioNanoScience.

[212] Kesharwani P., Ma R.Y., Sang L., Fatima M., Sheikh A., Abourehab M.A.S., Gupta N., Chen Z.S., Zhou Y. (2023). Gold nanoparticles and gold nanorods in the landscape of cancer therapy. Mol. Cancer.

[213] Kazemi Y., Dehghani S., Soltani F., Abnous K., Alibolandi M., Taghdisi S.M., Ramezani M. (2022). PNA-ATP aptamer-capped doxorubicin-loaded silica nanoparticles for targeted cancer therapy. Nanomedicine.

[214] Roy K., Kanwar R.K., Cheung C.H.A., Fleming C.L., Veedu R.N., Krishnakumar S., Kanwar J.R. (2015). Locked nucleic acid modified bi-specific aptamer-targeted nanoparticles carrying survivin antagonist towards effective colon cancer therapy. RSC Adv..

[215] Chen Z., Luo H., Gubu A., Yu S., Zhang H., Dai H., Zhang Y., Zhang B., Ma Y., Lu A. (2023). Chemically modified aptamers for improving binding affinity to the target proteins via enhanced non-covalent bonding. Front. Cell Dev. Biol..

[216] Liu M.P., Wang L., Lo Y., Shiu S.C.C., Kinghorn A.B., Tanner J.A. (2022). Aptamer-Enabled Nanomaterials for Therapeutics, Drug Targeting and Imaging. Cells.

[217] Zhou J.H., Rossi J. (2017). Aptamers as targeted therapeutics: Current potential and challenges. Nat. Rev. Drug Discov..

[218] Shi L., Wang L., Ma X., Fang X., Xiang L., Yi Y., Li J., Luo Z., Li G. (2021). Aptamer-Functionalized Nanochannels for One-Step Detection of SARS-CoV-2 in Samples from COVID-19 Patients. Anal. Chem..

[219] Jung H.H., Lee H., Yea J., Jang K.-I. (2024). Wearable electrochemical sensors for real-time monitoring in diabetes mellitus and associated complications. Soft Sci..

[220] Li J., Xiong M., Fu X.H., Fan Y., Dong C., Sun X., Zheng F., Wang S.W., Liu L., Xu M. (2023). Determining a multimodal aging clock in a cohort of Chinese women. Med.

[221] Tanaka T., Biancotto A., Moaddel R., Moore A.Z., Gonzalez-Freire M., Aon M.A., Candia J., Zhang P., Cheung F., Fantoni G. (2018). Plasma proteomic signature of age in healthy humans. Aging Cell.

[222] Zhavoronkov A., Mamoshina P. (2019). Deep Aging Clocks: The Emergence of AI-Based Biomarkers of Aging and Longevity. Trends Pharmacol. Sci..

[223] Froy O. (2011). Circadian rhythms, aging, and life span in mammals. Physiology.

[224] Cao Y.W., Wang R.H. (2017). Associations among Metabolism, Circadian Rhythm and Age-Associated Diseases. Aging Dis..

[225] Lee C., Etchegaray J.P., Cagampang F.R., Loudon A.S., Reppert S.M. (2001). Posttranslational mechanisms regulate the mammalian circadian clock. Cell.

[226] Zvonic S., Ptitsyn A.A., Conrad S.A., Scott L.K., Floyd Z.E., Kilroy G., Wu X., Goh B.C., Mynatt R.L., Gimble J.M. (2006). Characterization of peripheral circadian clocks in adipose tissues. Diabetes.

[227] Manoogian E.N.C., Panda S. (2017). Circadian rhythms, time-restricted feeding, and healthy aging. Ageing Res. Rev..

[228] Yeom J.W., Kim H., Pack S.P., Lee H.J., Cheong T., Cho C.H. (2025). Exploring the Psychological and Physiological Insights Through Digital Phenotyping by Analyzing the Discrepancies Between Subjective Insomnia Severity and Activity-Based Objective Sleep Measures: Observational Cohort Study. JMIR. Ment. Health.

[229] Nakazawa K., Matsuo M., Kikuchi Y., Nakajima Y., Numano R. (2024). Melanopsin DNA aptamers can regulate input signals of mammalian circadian rhythms by altering the phase of the molecular clock. Front. Neurosci..

